# A Numerical Study of the Microstructure-Dependent Thermal Conductivity of Diamond-Reinforced Sintered Silver Composites

**DOI:** 10.3390/ma19194246

**Published:** 2026-10-07

**Authors:** Zhengyang Tian, Jianxin Wu, Lunhu Hu, Yutai Su, Tiancai Cheng

**Affiliations:** 1 School of Mechanical Engineering, Inner Mongolia University of Technology, Hohhot 010051, China; 20251800021@imut.edu.cn (Z.T.); wujx@imut.edu.cn (J.W.); chengtc@imut.edu.cn (T.C.); 2 School of Mechanics and Transportation Engineering, Northwestern Polytechnical University, Xi’an 710129, China

**Keywords:** diamond, sintered silver, thermal interface material, representative volume element, finite element method, effective thermal conductivity, random microstructure

## Abstract

Diamond-reinforced sintered silver composites are considered promising thermal interface materials (TIMs) for power electronic packaging due to their combination of high thermal conductivity and mechanical reliability. This paper proposes and validates a numerical homogenization framework based on three-dimensional representative volume elements (RVEs) and steady-state finite element method (FEM) simulations to predict the effective thermal conductivity (k_eff_) of silver-diamond composites containing randomly distributed diamond particles and pores. Using a microstructure generation algorithm based on Gaussian random fields (GRF), a parametric study comprising 180 simulations was systematically conducted, covering variables such as porosity (10–50%), particle size (1–9 voxels), spatial distribution parameter σ, and diamond content. The results indicate that porosity is the dominant factor affecting k_eff_; within the studied parameter space, increasing porosity from 10% to 50% leads to a significant decrease in k_eff_ (approximately 40% in representative samples). Increasing diamond content significantly enhances k_eff_ under low-porosity conditions, but the benefit diminishes at high porosity levels, whereas k_eff_ correlates positively with increasing particle size. These findings highlight the critical role of microstructure design in achieving high-performance heat dissipation and provide practical guidance for optimizing diamond-sintered silver composite TIMs. The proposed three-dimensional random microstructure generation algorithm offers a viable approach for predicting the k_eff_ of such composite TIMs.

## 1. Introduction

With the rapid advancement of 5G communications, artificial intelligence, high-performance computing, and electric vehicles, modern electronic devices are evolving towards higher power density, miniaturized packaging, and multifunctional integration. This trend has led to a dramatic increase in the operating frequencies and power densities of electronic components, generating local heat flux densities reaching tens to hundreds of W/cm^2^ [1,2]. When heat within a device cannot be dissipated promptly, excessively high junction temperatures lead to reduced carrier mobility, degraded signal integrity, and accelerated material aging. In severe cases, this may even cause thermal failure of the device, rendering thermal management a critical bottleneck that constrains device reliability, performance, and lifespan. Consequently, efficient heat dissipation has become a core requirement for advanced electronic packaging [3,4,5]. Thermal interface materials (TIMs) are critical within the thermal management chain, as their thermal conductivity directly determines the upper limit of heat dissipation performance in electronic devices [6]. Thermal interface materials (TIMs) effectively reduce interfacial thermal resistance by filling micrometer-scale contact gaps between heat sources and heat sinks, thereby facilitating efficient heat transfer and preventing device overheating and thermal failure. However, conventional TIMs (such as thermal greases, phase change materials, and polymer-based thermal pads) struggle to meet the thermal management demands of next-generation wide bandgap power devices like gallium nitride (GaN) HEMTs and silicon carbide (SiC) MOSFETs [7,8]. This limitation stems from the low thermal conductivity of their polymer matrices and reliability issues such as pumping out or drying over extended use. In contrast, metal-based TIMs, particularly sintered silver (Ag) TIMs, have garnered significant attention due to their high intrinsic thermal conductivity (429 W·m^−1^·K^−1^), excellent electrical conductivity, and the ability to be sintered at low temperatures while maintaining operation at high temperatures [9,10].

Although highly dense sintered silver thermal interface materials (TIMs) have demonstrated exceptional mechanical strength and thermal fatigue resistance, pure metals or alloys struggle to simultaneously meet the synergistic demands of ideal thermal conductivity and coefficient of thermal expansion (CTE). Single-metal materials remain inadequate in addressing escalating heat-dissipation requirements. To further push the thermal performance limits of metal-based TIMs, incorporating ultra-high thermal conductivity ceramic reinforcing phases into the metallic matrix has emerged as a promising strategy [11,12,13]. Among these, diamond—the solid material with the highest thermal conductivity in nature (exceeding 2000 W·m^−1^·K^−1^ for high-purity type II single crystals at room temperature)—is regarded as an ideal reinforcing filler [14]. Experiments demonstrate that doped with 25 vol% of double-coated diamond particles (40–50 μm in size) into silver-based sintered composites yields a thermal conductivity of 243 W·m^−1^·K^−1^, representing an approximately 74% increase over undoped samples [15]. Consequently, diamond-reinforced sintered silver composites hold promise not only for achieving exceptional overall thermal conductivity but also for enhancing mechanical integrity by modulating the composite’s CTE [16,17]. However, the thermal conductivity of diamond/silver composites is highly dependent on complex microstructural parameters, including diamond particle size, content, spatial distribution, and matrix porosity. The coupled effects of these factors result in pronounced nonlinear thermal conduction behavior [18]. Research indicates that increasing diamond particle size substantially enhances the composite’s thermal conductivity. Concurrently, raising the filler volume fraction aids thermal conductivity improvement; however, measured thermal conductivities at high filler ratios often fall considerably below predictions from classical theoretical models [19]. Given that nanocomposite technology development demands molecular-level precision, purely experimental approaches are constrained by high costs and the difficulty of independently controlling microstructural parameters. Consequently, there is an urgent need to establish new controllable microstructure-generation methods and simulation frameworks to construct more realistic, highly filled metal matrix TIM microstructures for finite element thermal conduction simulations. Computer modeling serves as a powerful complement to experimental and analytical methods, enabling a deeper understanding of the multiscale behavior of complex materials [20]. Traditional analytical models typically rely on idealized assumptions (such as simplifying particles as spherical or regular shapes, uniform particle distribution, and a pore-free matrix), making it difficult to accurately describe the effects of actual model structures and pores. This represents one of the primary challenges to the accuracy of current models [21].

To overcome these limitations, microstructure-based numerical modeling (combining Representative Volume Element (RVE) and FEM simulations) has emerged as a powerful and flexible tool. For instance, Wenlong Tian et al. evaluated the effective thermal conductivity of discontinuous inclusion composites using an RVE-based finite element homogenization method with periodic boundary conditions [22]; Marcos-Gómez et al. investigated the influence of fiber anisotropy and interfacial thermal contact resistance on the overall thermal performance of CNF-reinforced copper-based composites through finite element calculations using RVEs [23]; Muhammad Owais Raza Siddiqui et al. employed finite element methods to predict the effective thermal conductivity and thermal resistance of fabrics [24]. However, idealized geometries and simplified interface configurations can compromise the accuracy of thermal conductivity predictions. Consequently, comprehensively evaluating the coupled effects of multiple structural factors—such as particle size, quantity, spatial arrangement, and porosity—on the thermal properties of composites remains a significant research priority [25]. In recent years, researchers have developed diverse methodologies to establish three-dimensional numerical models that authentically reflect the complex internal geometry and physical essence of composites, conducting sensitivity analyses on system parameters based on genuine three-dimensional microstructures. For instance, Elbana et al. employed spherical cavities and periodic RVE structures in their multiscale particle composite modeling research [26]; Xingwei Yan et al. proposed an RVE modeling strategy tailored for multiphase particles and core–shell hybrid composites [27]; while Chaocan Cai et al. generated RVEs for advanced composites using the maximum penetration bias algorithm [25]. Despite these advances in composite modeling, critical microstructural parameters and their coupled effects, alongside the influence of multiphase microstructures on thermal properties, warrant further investigation.

This study proposes a controllable RVE generation algorithm for evaluating the effective thermal conductivity of diamond-reinforced sintered silver composites. By integrating an enhanced Voronoi process, statistical filtering, and phase-separation techniques, this method enables independent control over diamond particle size, particle number, and silver matrix porosity within the composite. Based on the constructed three-phase microstructure, an RVE finite element homogenization computational framework was established. Steady-state heat conduction simulations were conducted to predict the equivalent thermal conductivity. A quantitative analysis was conducted on the influence of diamond particle size, diamond quantity, spatial structure, and matrix porosity on the effective thermal conductivity, revealing the interaction mechanisms among these factors. The proposed method holds promise for providing a reliable modeling basis and engineering references for the microstructural design of high-performance metal-based thermal interface materials (TIMs).

## 2. RVE Generation Method

### 2.1. Overview of RVE Generation Algorithm

This study proposes a method for generating a three-dimensional multiphase composite material microstructure RVE based on GRF and Gaussian filtering algorithms from image processing. The algorithm adopts a modular design, consisting of four core components: modified Voronoi segmentation, adaptive particle-size control, correlated random field generation, and threshold segmentation based on statistical quantiles. Input parameters include RVE geometry dimensions, target porosity, Gaussian filter width, number of diamond particles, and target diamond particle size.

**Figure 1:**
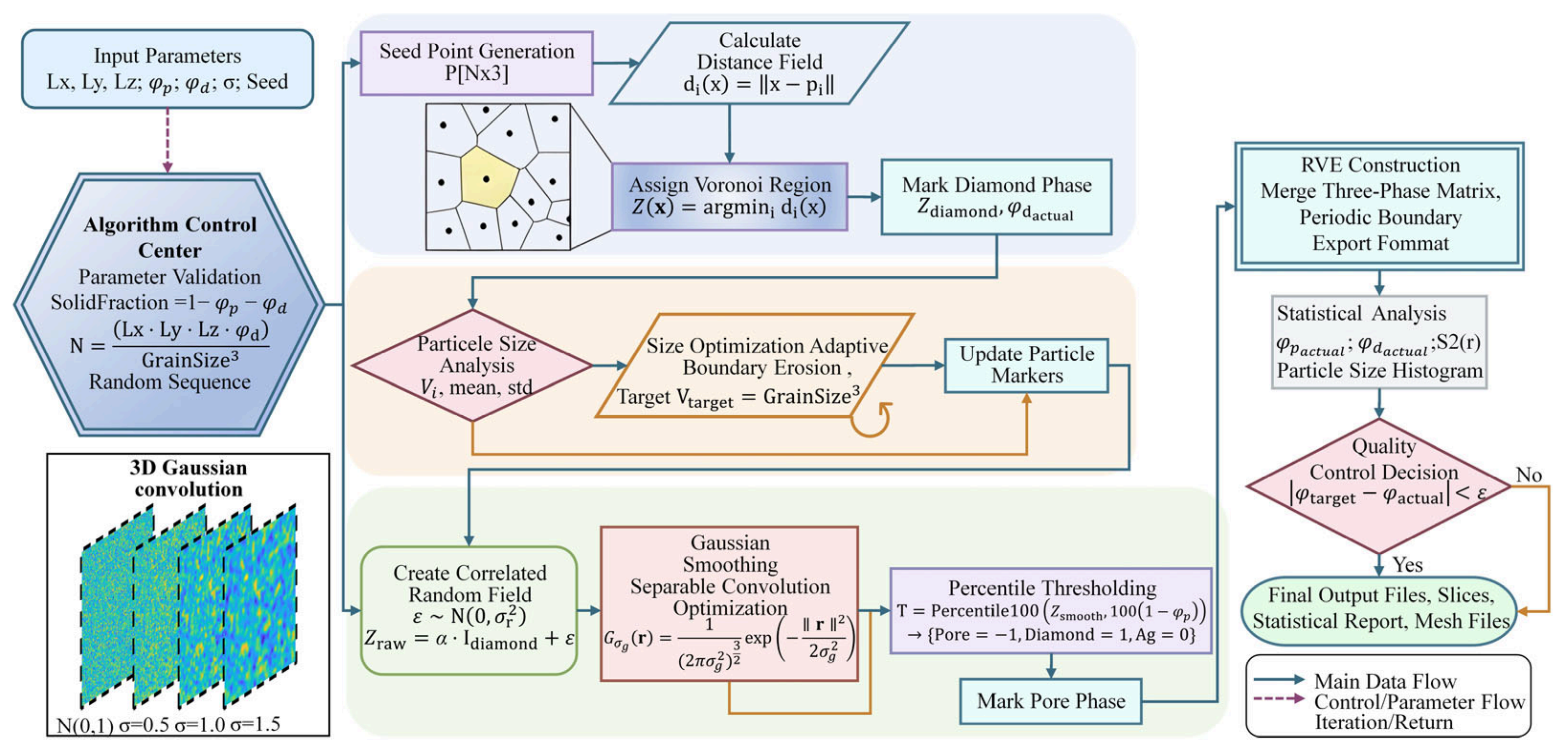
Algorithm workflow for generating a random microstructure composite material RVE.

The specific process is as follows: First, Voronoi seed points are uniformly arranged within the computational domain, and the Euclidean distance from each voxel to each seed point is calculated. Voxels are assigned to the Voronoi cell of the nearest seed to generate the initial diamond phase. Subsequently, an adaptive boundary erosion algorithm is applied to each particle to correct its size, approximating the target particle volume. Next, a random field modulated by the diamond distribution is constructed. After introducing spatial correlation via Gaussian filtering, threshold segmentation based on a quantile function is performed. This precisely extracts the pore phase while protecting the diamond phase, with the remaining region automatically defined as the silver matrix. The output is a discretized three-phase RVE microstructure with phase identification, enabling precise control over the pore phase, diamond phase, and silver matrix phase. It features adjustable statistical structural characteristics (such as two-point probability functions, particle size distribution) and compatibility with periodic boundary conditions. The detailed workflow of the algorithm is illustrated in Figure 1.

### 2.2. Composite Matrix Generation

The Voronoi diagram is a commonly used spatial partitioning method that divides space into multiple regions, each containing all points closest to a specific generating point (seed point). In materials science, Voronoi diagrams are used to simulate the grain structure of polycrystalline materials [26], the initial distribution of reinforcement phases in particle-reinforced composites [27], and RVE generation and particle distribution modeling [25], etc. Its mathematical definition for a set of discrete points in n-dimensional Euclidean space is

*P* = {*p*_1_,*p*_2_,…,*p_m_*} ⊂ *ℝ^n^* (1)

where *P_i_* = (x_i_, y_i_, z_i_) are the coordinates of the *i*-th seed point. The Voronoi region Vi corresponding to point Pi is defined as

*V_i_* = {**x** ∈ *ℝ^n^*∣*d*(**x**,**p***_i_*) ≤ *d*(**x**,**p***_j_*),∀*j* ≠ *i*} (2)

where **x** = (*x*,*y*,*z*) represents the coordinates of any point in space, and *d*(**x**,**p***_i_*) represents the Euclidean distance between point **x** and seed point **P**_i_.

To achieve the generation of the three-phase Diamond-Ag-Pore RVE, this study improves upon the traditional Voronoi diagram in the following aspects:

(a) Uniformity control: Ensures uniform distribution of diamond particles within the matrix, avoiding particle aggregation.
(b) Size controllability: Controls the consistency of particle size distribution through post-processing.
(c) Computational optimization: Utilizes vectorized operations to improve computational efficiency for large-scale three-dimensional grids.

First, the computational domain is defined as follows:

Ω = [0,*L_x_*] × [0,*L_y_*] × [0,*L_z_*] ⊂ *ℝ*^3^ (3)

where Lx, Ly, Lz are the dimensions of the computational domain in the x, y, and z directions, respectively. The computational domain is then partitioned according to the following equation:

(4) $$\Omega_{i,j,k}=\left[\frac{(i-1)L_{x}}{n_{x}},\frac{iL_{x}}{n_{x}}\right]\times \left[\frac{(j-1)L_{y}}{n_{y}},\frac{jL_{y}}{n_{y}}\right]\times \left[\frac{(k-1)L_{z}}{n_{z}},\frac{kL_{z}}{n_{z}}\right]$$

where **Ω***_i_*_,*j*,*k*_ denotes the *i*, *j*, *k*-th subregion, which is a three-dimensional rectangular parallelepiped; *i* = 1,2,…,*n*, *j* = 1,2,…,*n_y_*, *k* = 1,2,…,*n_z_*; *n_x_* × *n_y_* × *n_z_* is the number of subregions in each direction, controlling the spatial distribution density of diamond particles.m represents the number of initial seed points. To ensure *m* = *n_x_* × *n_y_* × *n_z_* and an approximately uniform spatial distribution, one seed point is randomly generated within each subregion **Ω***_i_*_,*j*,*k*_:

**P***_i_*_,*j*,*k*_ = (**x**_i,j,k_,**y**_i,j,k_,**z**_i,j,k_) (5)

where the coordinate components follow a uniform distribution:

(6) $$\begin{array}{c} x_{i,j,k}∼u\left(\frac{(i-1)L_{x}}{n_{x}},\frac{iL_{x}}{n_{x}}\right)\\ y_{i,j,k}∼u\left(\frac{(j-1)L_{y}}{n_{y}},\frac{jL_{y}}{n_{y}}\right)\\ z_{i,j,k}∼u\left(\frac{(k-1)L_{z}}{n_{z}},\frac{kL_{z}}{n_{z}}\right) \end{array}$$

Subsequently, the continuous computational domain Ω is discretized into a regular three-dimensional voxel grid according to the following equation:

𝒢 = {(*x_l_*,*y_m_*,*z_n_*)∣*l* = 1,⋯,*N_x_*; *m* = 1,⋯,*N*; *n* = 1,⋯,*N_z_*} (7)

(8) $$\begin{array}{c} x_{l}=(1-0.5)\Delta x,\Delta x=\frac{L_{x}}{N_{x}}\\ y_{m}=(m-0.5)\Delta y,\Delta y=\frac{L_{y}}{N_{y}}\\ z_{n}=(n-0.5)\Delta z,\Delta z=\frac{L_{z}}{N_{z}} \end{array}$$

where *N_X_*, *N_y_*, *N_z_* represent the number of voxels in each direction; Δ_x_, Δ_y_, Δ_z_ represent the voxel dimensions in the x, y, z directions; (*x_l_*, *y_m_*, *z_n_*) represent the coordinates of the voxel center point. For each voxel (*x_l_*, y*_m_*, *z_n_*), the Euclidean distance to all seed points is calculated according to the following equation:

(9) $$d_{i,m,n}^{\left(i,j,k\right)}=\sqrt{{\left(x_{i}-x_{i,j,k}\right)}^{2}+{\left(y_{m}-y_{i,j,k}\right)}^{2}+{\left(z_{n}-z_{i,j,k}\right)}^{2}}$$

Finally, based on the above work, Voronoi assignment is performed. Each voxel is assigned to the Voronoi cell of the nearest seed point according to the following equation:

(10) $${\mathrm{Voronoi}}_{\mathrm{label}}(l,m,n)=\arg\underset{\left(i,j,k\right)}{\min}d_{l,m,n}^{\left(i,j,k\right)}$$

(11) $$Z(l,m,n)=\{(i,j,k)|d_{l,m,n}^{\left(i,j,k\right)}\leq d_{l,m,n}^{\left(i,j^{\prime },k^{\prime }\right)},\forall (i^{\prime },j^{\prime },k^{\prime })\neq (i,j,k)\}$$

where arg min returns the argument that minimizes the distance function; Z(*l*,*m*,*n*) represents the Voronoi cell label to which the voxel (*l*,*m*,*n*) belongs. At this stage, the label corresponds to a diamond.

The above steps enable the Voronoi cells to automatically fill the entire space. Simultaneously, the particle distribution generated by the Voronoi diagram exhibits statistical randomness, simulating the non-periodic distribution of the reinforcement phase in real materials. The volume fraction of the diamond phase is controlled by adjusting the seed point density. However, the initial Voronoi segmentation yields a relatively wide particle size distribution. To achieve uniform particle sizes, a size control algorithm needs to be introduced.

### 2.3. Particle Size Control Algorithm

To adjust particle sizes to target volumes while maintaining the physical rationality of particle morphology, this paper proposes an adaptive boundary erosion algorithm. This algorithm takes the Voronoi label matrix Z_diamond_ as input and outputs the size-adjusted label matrix Z′_diamond_.

First, the discrete volume *V_i_* of the *i*-th Voronoi region is calculated:

(12) $$V_{i}=\sum_{x\in \Omega }I(Z(x)=i)\cdot \nu_{0}$$

where x = (*x*,*y*,*z*) represents the voxel coordinates in three-dimensional space; **Ω** is the computational domain, **Ω** = [0,*L_x_*] × [0,*L_y_*] × [0,*L_z_*]; Z(**x**) is the Voronoi label of voxel **x**; 𝕀(⋅) represents the indicator function, which equals 1 if the condition is true, and 0 otherwise; *v*_0_ is the volume of a single voxel, *ν*_0_ = Δ*x*⋅Δ*y*⋅Δ*z*.

Then, a uniformity metric for particle size distribution is defined as the relative variance of volumes:

(13) $$J(\{V_{i}\})=\frac{1}{n}\sum_{i=1}^{n}{\left(\frac{V_{i}-\bar{V}}{\bar{V}}\right)}^{2},\bar{V}=\frac{1}{n}\sum_{i=1}^{n}V_{i}$$

where *n* represents the total number of particles; $\bar{V}$ represents the average particle size; *V_i_* represents the volume of the *i*-th particle.

Finally, the algorithm minimizes *J* through iterative erosion operations, while keeping the total diamond volume fraction approximately constant. The diamond volume fraction is controlled via the following equation:

(14) $$\phi_{d}\approx \frac{m\cdot \bar{V}}{L_{x}L_{y}L_{z}}$$

The core steps of adaptive boundary erosion are as follows: For each particle, the algorithm identifies its surface voxels (i.e., voxels that have at least one face adjacent to a voxel with a different label). In each iteration, a probability *p_erode_* is calculated based on the ratio of the current particle volume *V_i_* to the target volume *V_target_* (which can be set to $\bar{V}$ or a preset value). For surface voxels, they are removed from the current particle (label set to 0) with probability *p_erode_*. This process is iterated until all particle volumes are close to the target volume, or the maximum number of iterations is reached. The eroded voxels will be considered part of the silver matrix in subsequent steps. Finally, all adjusted particle voxel labels are uniformly set to 1, obtaining a binary distribution matrix *Z_diamond_* for the diamond phase. This step achieves preliminary control over diamond particle size and reserves space for the subsequent generation of the pore phase. The algorithm pseudocode is shown in Algorithm 1.
**Algorithm 1: Adaptive Boundary Erosion**1: for i = 1 to n do2:     Calculate current volume V*_i_* = |{x: Z(x) = i}|3:     if V*_i_* ≤ V_target_ then4:         Continue to next region5:     else6:         Initialize R*_i_* = {x: Z(x) = i}7:         t = 08:         while |R*_i_*| > V_target_ and t < T_max_ do9:             Calculate boundary voxel set B_i_ = {x ∈ R_i_: ∃ y ∈ N(x), Z(y) ≠ i}10:            if B*_i_* is empty then11:                break loop12:            end if13:            Randomly select and remove a subset ΔB*_i_* from B*_i_*, satisfying |ΔB*_i_*| = min(ρ|B*_i_*|, |R*_i_*|-V_target_)14:            Update R*_i_* = R*_i_* \ ΔB*_i_*15:            t = t + 116:        end while17:        Update Z′[R*_i_*] = i18:    end if19: end

### 2.4. Pore Phase Generation Based on Gaussian Random Field and Quantile Cutting

The pore distribution within the sintered silver matrix exhibits randomness and spatial correlation. To generate pores with specific volume fractions and morphological characteristics within the remaining region (the silver matrix region) based on the already generated diamond phase, this algorithm introduces a Gaussian Random Field (GRF) model.

First, an initial random field X(x) correlated with the diamond distribution is generated over the entire RVE domain. Specifically, different assignment strategies are employed in the diamond phase region (*Z*_diamond_ = 1) and the candidate silver matrix region (*Z*_diamond_ = 0):

(15) $$X_{0}(x)=\left\{\begin{array}{c} c_{\mathrm{diamond}},\;\mathrm{if}\;Z_{\mathrm{diamond}}(x)=1\\ N(0,1),\;\mathrm{if}\;Z_{\mathrm{diamond}}(x)=0 \end{array}\right.$$

where *c*_diamond_ is a constant significantly larger than typical values of a Gaussian distribution, intended to protect the diamond phase from being misclassified as pores in subsequent processing; 𝒩(0,1) represents independent random sampling from a standard normal distribution.

To simulate the spatial correlation of pore formation (i.e., pores tend to form clusters of limited size rather than being completely random scatter points), a three-dimensional Gaussian filter is applied to the initial random field *X_0_*:

*X*_filtered_ = *G_σ_* ⋅ *X*_0_ (16)

(17) $$G_{\sigma }(x,y,z)=\frac{1}{{\left(2\pi \sigma^{2}\right)}^{3/2}}\exp\left(-\frac{x^{2}+y^{2}+z^{2}}{2\sigma^{2}}\right)$$

where *G_σ_* is a three-dimensional isotropic Gaussian convolution kernel with standard deviation σ. The physical significance of Gaussian filtering is that it makes the value at each point influenced by random values within its neighborhood. The filter width σ directly controls the characteristic size of the final pores: the larger the σ, the stronger the pore connectivity and the larger the average pore size.

Subsequently, a threshold segmentation method based on the quantile function is employed to extract the pore phase with a specific volume fraction *p* (porosity) from the continuous filtered random field *X*_filtered_. First, the values of all voxels in *X*_filtered_ are flattened into a one-dimensional array *X*_flat_, ignoring the diamond phase region (whose values have been set to the constant *c*_diamond_, typically at the extreme end of the distribution). The empirical quantile function Q(*ϕ*) of this array is calculated. It provides, for a given volume fraction *ϕ*, the corresponding threshold t such that the proportion of voxels with values less than t is approximately *ϕ*. The target porosity corresponds to a quantile level *ϕ* = *p* ⋅ (1 − *ϕ_d_*), because pores can only originate from the non-diamond phase (which has a volume fraction of 1 − *ϕ_d_*). The corresponding cutting threshold *t_p_* is calculated:

*t_p_* = *Q*(*ϕ*) (18)

Then, threshold segmentation is applied to define the pore phase *Z*_pore_ and the silver matrix phase *Z*_Ag_:

(19) $$\begin{array}{c} Z_{\mathrm{pore}}(x)=1\;\mathrm{if}\;X_{\mathrm{filtered}}(x)<t_{p}\;\mathrm{and}\;Z_{\mathrm{diamond}}(x)=0\\ Z_{\mathrm{Ag}}(x)=1\;\;\mathrm{if}\;X_{\mathrm{filtered}}(x)\geq t_{p}\;\mathrm{and}\;Z_{\mathrm{diamond}}(x)=0 \end{array}$$

Finally, the three-phase RVE is completely defined by the following label matrix:

(20) $$Z_{\mathrm{RVE}}(x)=\left\{\begin{array}{ccc} 1 & (\mathrm{Diamond}), & \mathrm{if}\;Z_{\mathrm{diamond}}(x)=1\\ 0 & (\mathrm{Ag}), & \mathrm{if}\;Z_{\mathrm{Ag}}(x)=1\\ -1 & (\mathrm{Pore}), & \mathrm{if}\;Z_{\mathrm{pore}}(x)=1 \end{array}\right.$$

**Figure 2:**
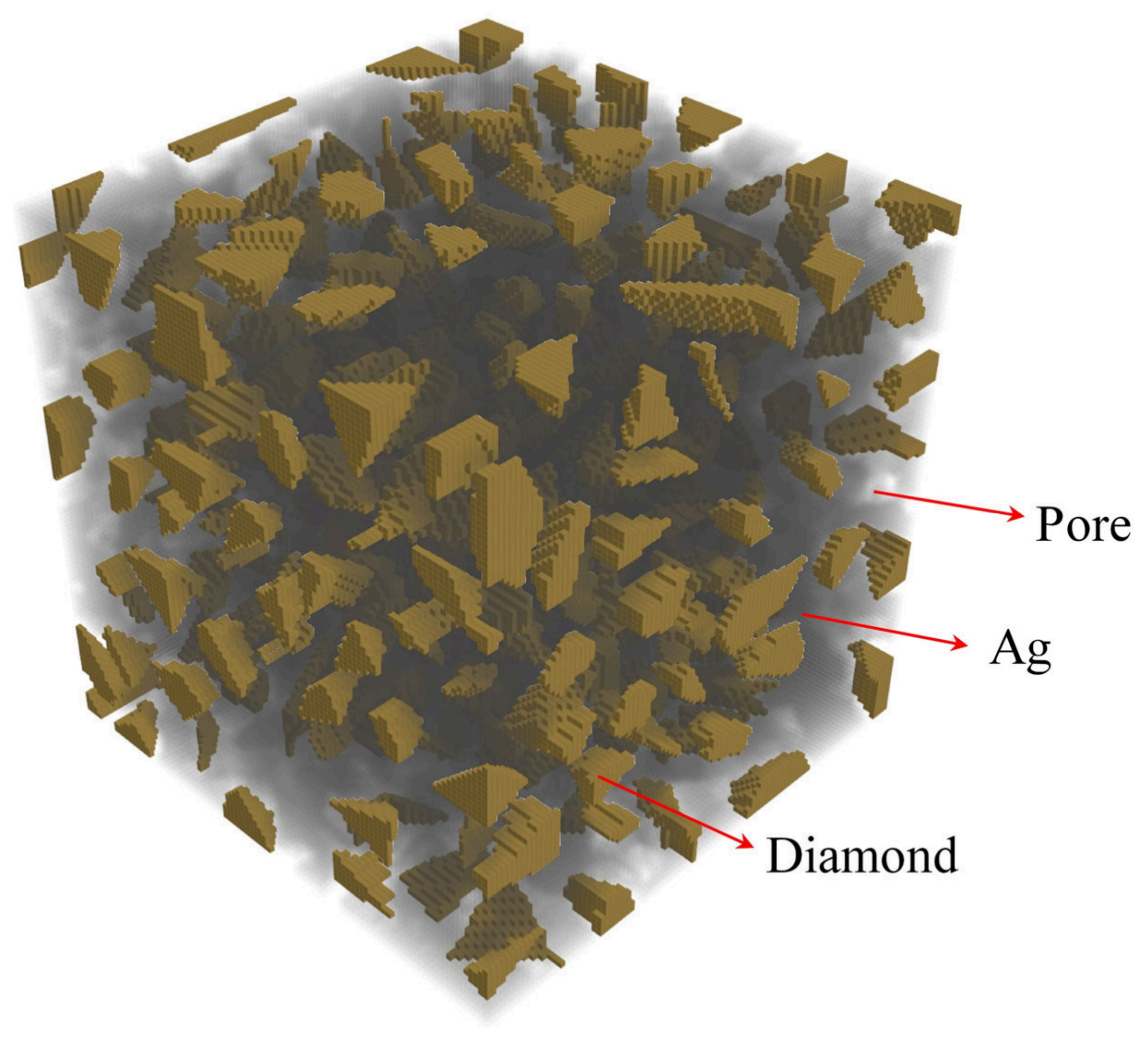
Illustration of RVE model components.

Figure 2 illustrates a generated RVE. The model size is 100 × 100 × 100 (the model size is fixed to this value in the following sections), with a porosity of 25%, a Gaussian kernel width of 2, the number of reinforcing diamond phases is 7 × 7 × 7, and the diamond particle size is 7. In the figure, the collection of yellowish-brown cubes represents diamond, the collection of gray cubes represents silver, and the remaining collection of white cubes represents pores.

In the present microstructure-generation framework, the parameter *p* is defined as a nominal pore-generation parameter that controls the quantile-cut threshold of the filtered random field. It should not be interpreted as the exact pore volume fraction of the final three-phase RVE. The actual pore volume fraction is obtained a posteriori from the generated voxelized microstructure.

Based on the three-phase composite material RVE generated above with controllable microstructural features, the next chapter will fix the model size and elaborate on the parameter assignment method concerning different porosities, diamond volume fractions, particle sizes, and different Gaussian kernel widths for the three-phase composite. A finite element homogenization analysis framework will be established to predict the macroscopic thermal conductivity from the microstructure.

It should be noted that the present generator explicitly introduces tunable spatial correlation only for the pore phase through Gaussian filtering. By contrast, the diamond phase is initialized by approximately uniform seed placement followed by size adjustment; therefore, the model does not explicitly simulate particle agglomeration in the strict physical sense.

## 3. Finite Element Homogenization Method for Macroscopic Thermal Conductivity Based on RVE

### 3.1. Heat Transport Mechanism and Constitutive Parameters

The core function of a TIM is to construct an efficient heat transfer channel between two solid surfaces, thereby minimizing the interfacial thermal contact resistance. Under certain packaging pressure and operating temperature conditions, the overall thermal resistance of the TIM layer is primarily composed of two parts: the bulk thermal resistance of the material itself (thickness divided by thermal conductivity) and the interfacial thermal contact resistance at the two ends. For micro-/nano-scale TIM layers, the bulk thermal resistance often dominates, and this bulk resistance is directly determined by the material’s thermal conductivity. Therefore, enhancing the material’s *k_eff_* is a crucial pathway to improving the heat transfer performance of TIMs. This study aims to establish a predictive model connecting microstructure to macroscopic heat transport performance. The thermal conductivity, as a constitutive parameter describing the macroscopic heat-transport capability, serves as the most direct bridge between the two.

In the diamond-silver three-phase composite material, its *k_eff_* is influenced by the complex coupling of multiple microstructural parameters (to be discussed in detail in subsequent sections), and the thermal conductivities of each phase differ significantly. Diamond, as an ultra-high thermal conductivity reinforcement phase, adopts its typical intrinsic thermal conductivity value: *k*_diamond_ = 2000W/(m ⋅ K). The sintered silver matrix is discretized within the RVE into a dense silver phase and a pore phase. The dense silver phase, as a high thermal conductivity matrix, has a thermal conductivity of: *k*_Ag_ = 420W/(m ⋅ K). The pores, on the other hand, act as a nearly adiabatic phase, filled with stagnant air, with a thermal conductivity of: *k_p_*_ore_ = 0.023W/(m ⋅ K). The thermophysical properties of these three phases collectively determine the complex macroscopic heat transport behavior of the three-phase composite material.

### 3.2. Governing Equation for Steady-State Heat Conduction and Finite Element Discretization

After clearly defining the thermophysical property parameters of the three phases, it is necessary to establish the governing equation describing heat transfer within this complex microstructure and transform it into a solvable numerical model. For a steady-state heat conduction problem with no internal heat source and constant material properties, the temperature field T(x) within the RVE domain **Ω** satisfies the Laplace equation based on energy conservation:

∇⋅[*k*(**x**)∇*T*(**x**)] = 0, ∀**x** ∈ Ω (21)

where k(x) is the spatially varying local thermal conductivity, directly determined by the phase to which the voxel belongs. In the diamond region **Ω***_d_*, k(x) = k*_d_*; in the silver matrix region **Ω***_Ag_*, k(x) = k*_Ag_*; in the pore region **Ω***_p_*, k(x) = k*_d_*. Due to the random distribution of the three phases, an analytical solution to Equation (21) is unattainable, necessitating the use of numerical methods.

To ensure accurate capture of phase interface geometry and to adapt the front-end voxel-based generation algorithm, this paper employs a regular voxel-based finite element method. The computational domain **Ω** is discretized into *N_x_* × *N_y_* × *N_z_* cubic elements, with uniform material properties assigned within each element. Each cube is further divided into an eight-node hexahedral isoparametric element, DC3D8. The temperature field *T*^(*e*)^ within an element is interpolated from the nodal temperatures *T*^(*e*)^ using shape functions N:

(22) $$T^{\left(e\right)}(\xi ,\eta ,\zeta )=N(\xi ,\eta ,\zeta )T^{\left(e\right)}=\sum_{i=1}^{8}N_{i}(\xi ,\eta ,\zeta )T_{i}^{\left(e\right)}$$

where (*ξ*,*η*,*ζ*) represents the local coordinates defined on the standard cube [−1,1]^3^.

Applying the Galerkin weighted residual method to the governing Equation (21) and integrating over each element domain **Ω***_e_* yields the element heat conduction matrix K^(*e*)^:

(23) $$K^{\left(e\right)}=\int_{-1}^{1}\int_{-1}^{1}\int_{-1}^{1}B^{T}k^{\left(e\right)}B\left|J\right|\mathrm{d\xi d\eta d\zeta}$$

where **B** = ∇**N** is the shape function gradient matrix, relating nodal temperatures to the temperature gradient within the element; *k*^(*e*)^ is the material thermal conductivity of that element; |**J**| is the Jacobian determinant for the mapping from local to global coordinates, which is constant for the regular cubic mesh used in this study. The *k*^(*e*)^ matrices for all elements are assembled according to global node numbers to form the global heat conduction matrix **K**, ultimately resulting in a large sparse system of linear equations:

**KT** = **F** (24)

where **T** is the global nodal temperature vector, and **F** is the nodal heat flux load vector, determined by the applied boundary conditions.

### 3.3. One-Dimensional Heat Flux Driving and Fixed Temperature Boundary Conditions

**Figure 3:**
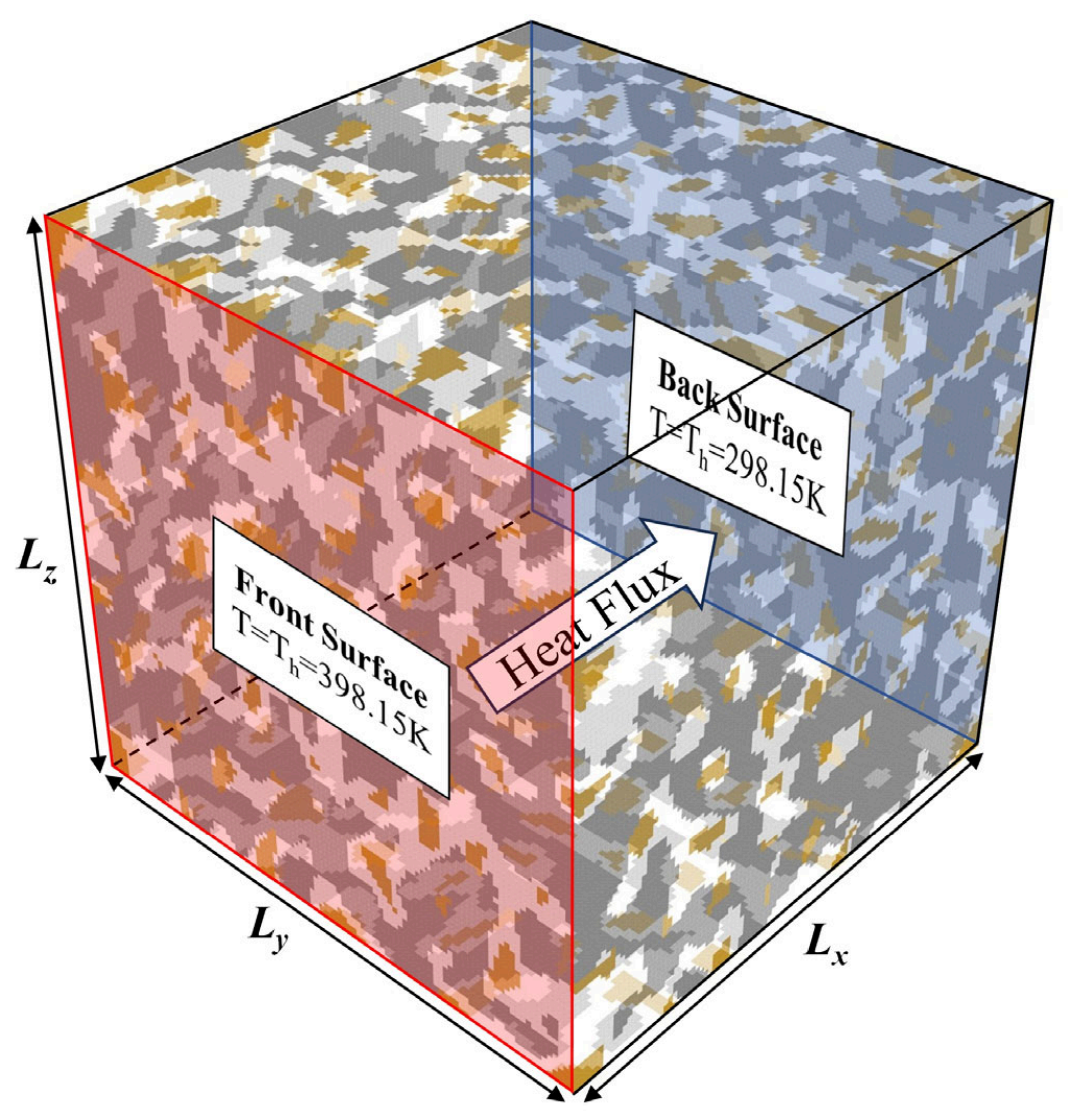
Fixed temperature boundary conditions.

To compute the macroscopic equivalent thermal conductivity of the three-phase composite material in a specific direction, appropriate boundary conditions must be applied to the RVE to simulate steady-state heat conduction. In actual electronic packaging, the TIM layer is typically sandwiched between two approximately isothermal solid surfaces, forming a one-dimensional heat conduction path dominated by the direction perpendicular to the interfaces. Therefore, this study adopts Dirichlet boundary conditions to drive the macroscopic heat flux. As illustrated in Figure 3, a high temperature *T_h_* = 398.15 K, is applied to all nodes on the front surface (*x* = 0) of the RVE, and a low temperature *T_c_* = 298.15 K is applied to all nodes on the back surface (*x* = *L_x_*), establishing a constant macroscopic temperature gradient:

(25) $$\nabla T_{x}=\frac{T_{c}-T_{h}}{L_{x}}=-\frac{\Delta T}{L_{x}}=-100K/m$$

where Δ*T* = 100 K. The remaining four surfaces were treated as adiabatic boundaries with no applied heat flux. This suppresses lateral heat leakage and approximates the one-dimensional through-thickness heat-transfer condition of a thermal interface material operating between two nearly isothermal solid surfaces. In this case, the normal heat flux q⋅n = 0.

### 3.4. Macroscopic Heat Flux Statistics and Effective Thermal Conductivity Extraction Method

After obtaining the microscopic heat flux field satisfying the boundary conditions, it is finally necessary to extract the *k_eff_* representing the properties of the macroscopic homogeneous material. According to Fourier’s law, the macroscopic *k_eff_* is defined as the ratio of the macroscopic heat flux density ${\bar{q}}_{x}$ to the applied macroscopic temperature gradient ∇*T_x_*:

(26) $$k_{\mathrm{eff}}=-\frac{{\bar{q}}_{x}}{\nabla T_{x}}$$

The macroscopic heat flux density ${\bar{q}}_{x}$ is the volume average of the microscopic heat flux field *q_x_*(**x**) over the RVE:

(27) $${\bar{q}}_{x}=\frac{1}{V}\int_{\Omega }q_{x}(x)\mathrm{dV}$$

Since the present RVE is discretized into regular voxel elements with equal volumes, the above expression can be written in the following discrete form:

(28) $${\bar{q}}_{x}\approx \frac{1}{N_{\mathrm{tot}}}\sum_{e=1}^{N_{\mathrm{tot}}}q_{x,e}$$

where *N*_tot_ is the total number of finite elements in the entire three-phase model, and *q_x_*_,*e*_ denotes the *x*-direction component of the heat flux in the *e*-th element. In the post-processing procedure, the elemental heat flux is extracted from all elements belonging to the diamond, silver, and pore phases, and the macroscopic heat flux is determined from the volume average over the entire computational domain. For mechanistic analysis only, the phase-averaged heat fluxes of the diamond, silver, and pore phases can also be evaluated separately:

(29) $${\langle {\bar{q}}_{x}\rangle }_{m}=\frac{1}{V_{m}}\int_{\Omega }q_{x}(x)dV\approx \frac{1}{N_{m}}\sum_{e=1}^{N_{m}}q_{x,e}^{\left(m\right)},\;m\in \{d,\mathrm{Ag},p\}$$

where *V_m_* and *N_m_* denote the volume and the number of elements of phase *m*, respectively. These phase-wise averages are used only to analyze phase-level heat-conduction characteristics. They cannot be used directly as substitutes for the macroscopic heat flux in the calculation of effective thermal conductivity. Therefore, the final expression used for the effective thermal conductivity in this study is

(30) $$k_{\mathrm{eff},x}=\frac{L_{x}}{\Delta T}\left|\frac{1}{N_{\mathrm{tot}}}\sum_{e=1}^{N_{\mathrm{tot}}}q_{x,e}\right|$$

## 4. Discussion

### 4.1. Microstructural Characterization of Generated RVEs

Under the premise of a constant RVE material size, the diamond volume fraction is jointly determined by the number of diamond particles and their size. To more comprehensively reveal the influence of the diamond reinforcement phase, this study controls four key structural parameters—porosity, number of diamond particles, diamond particle size, and Gaussian kernel width—based on the methods described in Chapters 2 and 3. The dataset comprises a total of 180 independent simulation results from a 3 × 4 × 5 × 3 factorial combination, covering porosity (10%, 30%, 50%), number of diamond particles (27, 125, 343, 729), diamond particle size (1, 3, 5, 7, 9 voxels), and Gaussian kernel width (1.0, 1.5, 2.0). It should be noted that the number of diamond particles corresponds to the number of initial seed points *m* = *n_x_* × *n_y_* × *n_z_* mentioned in Chapter 2, where *n_x_* = *n_y_* = *n_z_* = *n* and *m* = *n*^3^ are used. All simulations utilized the described procedural workflow to generate numerous INP files, which were submitted to Abaqus for computation, with thermal conductivity results automatically extracted via post-processing scripts.

The actual phase volume fractions deviate to some extent from the nominal parameter values specified in the manuscript. For ease of presentation, the following discussion still uses the nominal parameter names. The corresponding actual phase volume fractions for all parameter combinations, together with the associated thermal-conductivity results, have been made publicly available in the authors’ GitHub repository. The link is as follows: https://github.com/lucifertianzhengyang-netizen/Thermal-conductivity-simulation-analysis-data-of-diamond-sintered-nano-silver-RVEes-RVE.git (6 April 2026).

**Figure 4:**
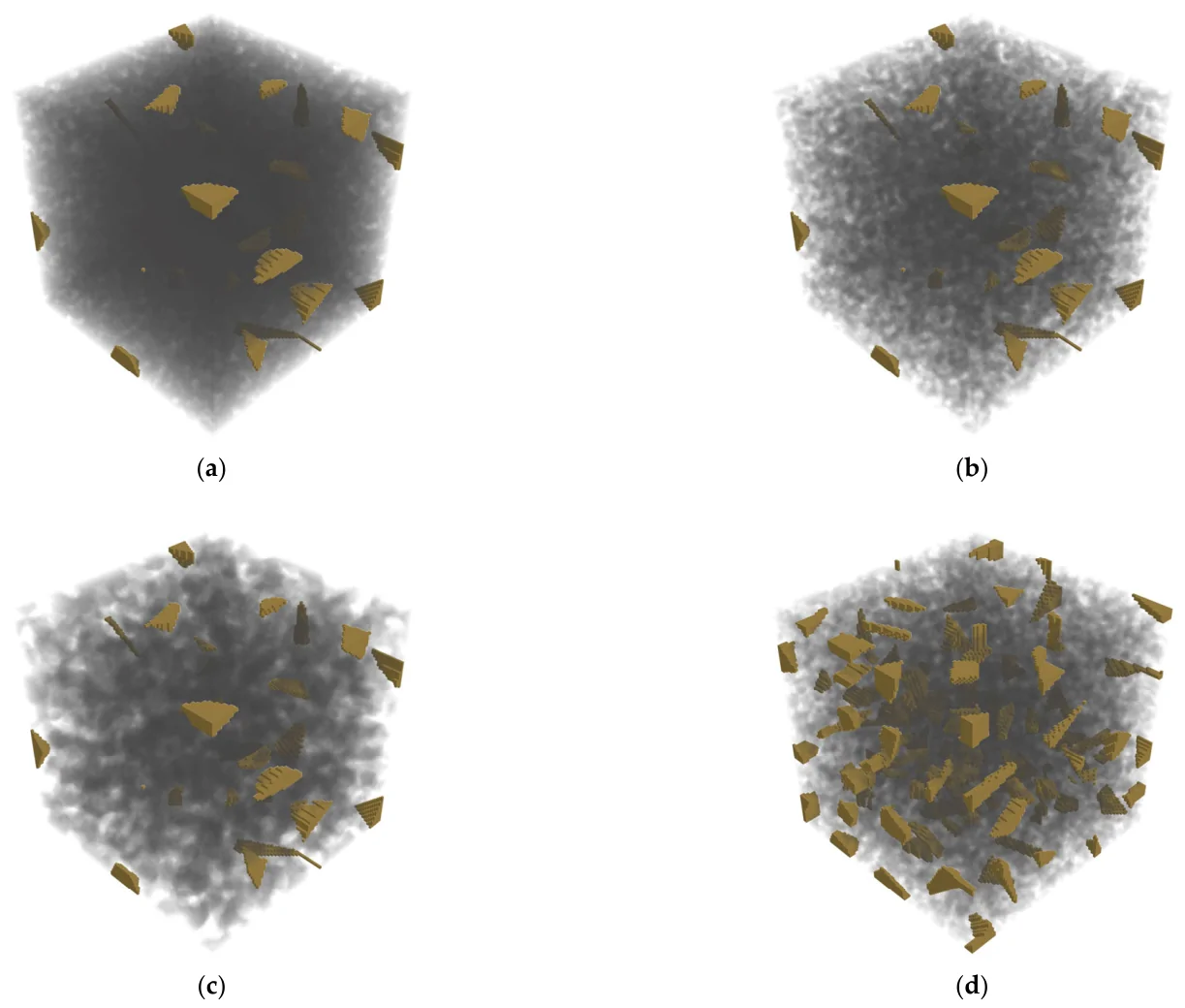

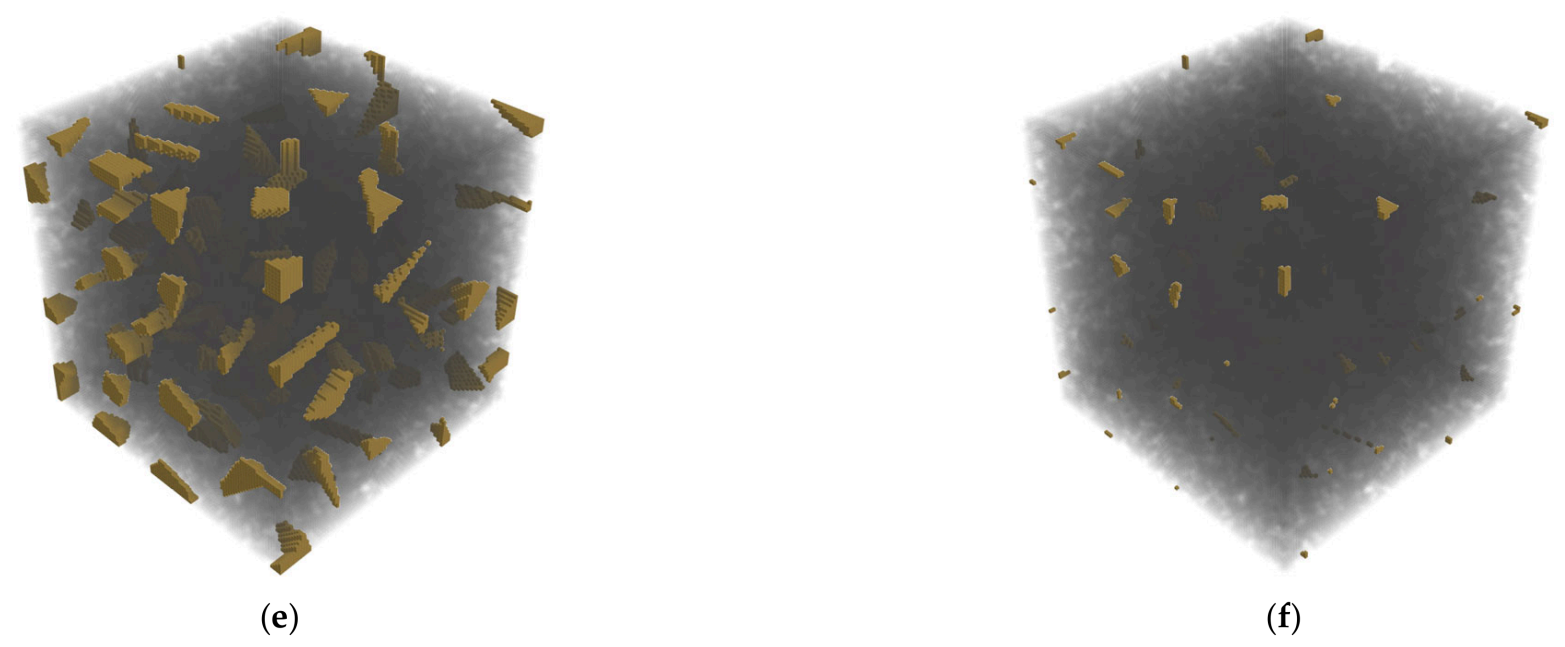
RVE microstructural representations under six parameter combinations: (**a**) 10%_1.0_27_7, (**b**) 50%_1.0_27_7, (**c**) 50%_2.0_27_7, (**d**) 50%_1.0_125_7, (**e**) 10%_1.0_125_7, (**f**) 10%_1.0_125_3.

The focus of this study is to elucidate the trends and mechanisms associated with relative size variations. In the program and all subsequent discussions, diamond particle size and Gaussian kernel width are expressed in units of voxels. This is a relative length unit corresponding to the mesh size after RVE discretization. Figure 4a–f display the RVE microstructural representations under different parameter combinations of porosity, σ, diamond particle size, and number of diamond particles. Comparing Figure 4a,b and Figure 4b,d, it can be observed that porosity and the number of diamond particles primarily influence the volume fractions of each phase. Comparing Figure 4e,f and Figure 4b,c, it is evident that diamond particle size and Gaussian kernel width mainly affect the morphological characteristics of each phase. Therefore, subsequent subsections will discuss the influence of variations in these four parameters on thermal conductivity from these two aspects.

### 4.2. Influence of Phase Content on Effective Thermal Conductivity

In three-phase composites, the volume distribution of each phase exerts a dominant influence on their thermal conduction performance. In diamond-reinforced sintered silver composites, porosity and the number of diamond particles control the volume fractions of the low-thermal-conductivity phase (pores) and the high-thermal-conductivity reinforcement phase (diamond) within the composite, respectively. This set of studies fixes the diamond size and σ to investigate the effects of porosity and the number of diamond particles on *k_eff_*.

**Figure 5:**
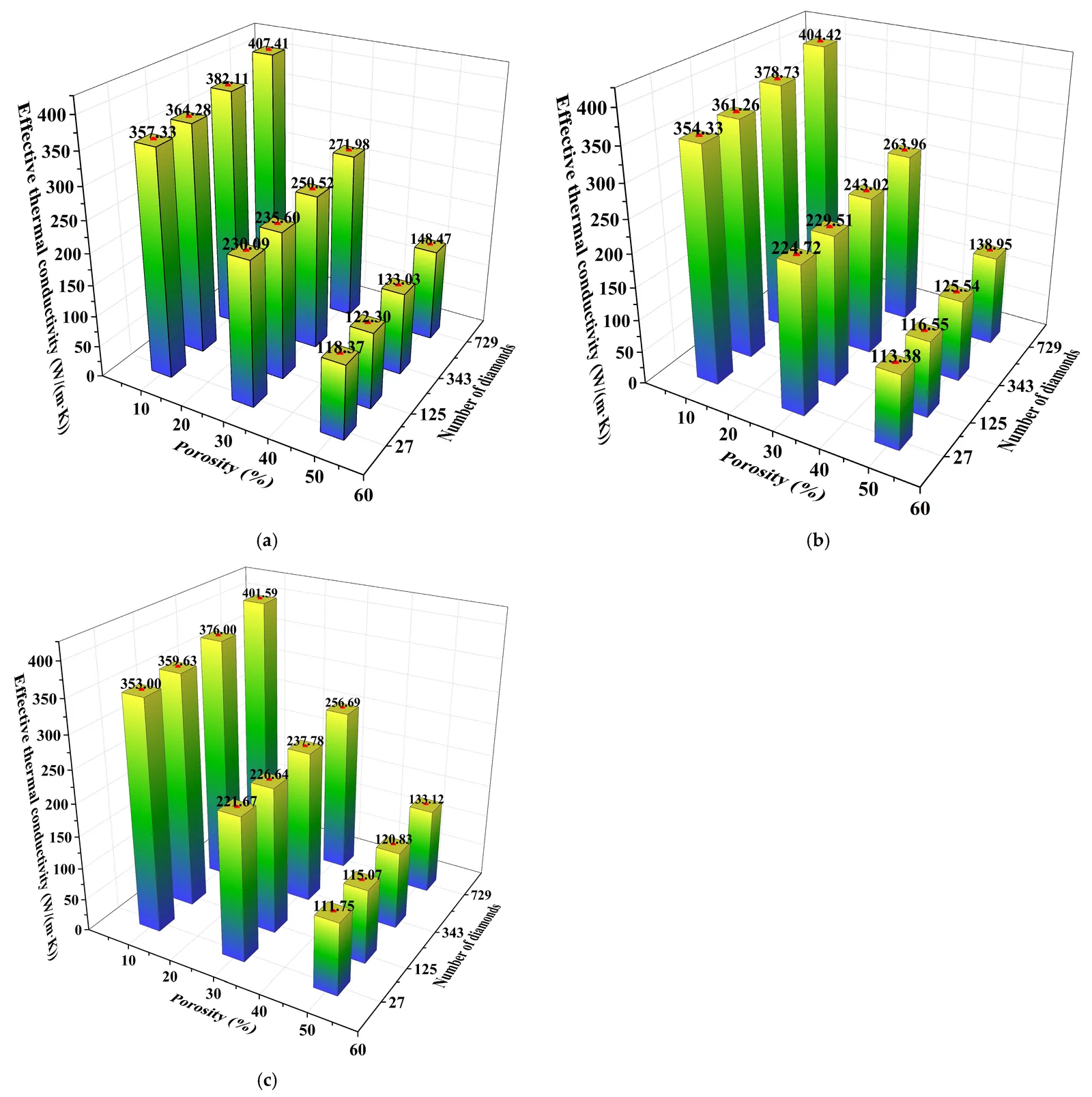
Bar charts of *k_e__ff_* variation under three σ values: (**a**) σ = 1.0, (**b**) σ = 1.5, (**c**) σ = 2.0.

Figure 5a–c show bar charts of *k_eff_* as a function of porosity for different numbers of diamond particles, with diamond size fixed at 5 and σ ranging from 1.0 to 2.0. It can be observed from the figures that, across different combinations of σ and diamond particle number, *k_eff_* is negatively correlated with porosity. Taking Figure 5a as an example, when the number of diamond particles is 27, a 40% increase in porosity leads to a 40.43% decrease in *k_eff_*. Analysis of the data confirms that this trend holds true for different diamond particle sizes.

This significant decrease arises because pores act as thermal resistance regions, obstructing the conduction paths for phonons and electrons. This forces heat flow to detour, disrupts the spatial continuity of the silver phase, reduces the effective cross-section for thermal conduction, and produces a blocking effect, thus leading to a sharp decline in thermal conductivity.

It can also be seen from the figures that, across different combinations of σ and porosity, *k_eff_* is positively correlated with the number of diamond particles. Analysis of the data confirms that this trend holds true for different diamond particle sizes. This trend occurs because diamond, as a high-thermal-conductivity reinforcement phase, increases the density of high-thermal-conductivity pathways within the composite material as its number increases.

**Figure 6:**
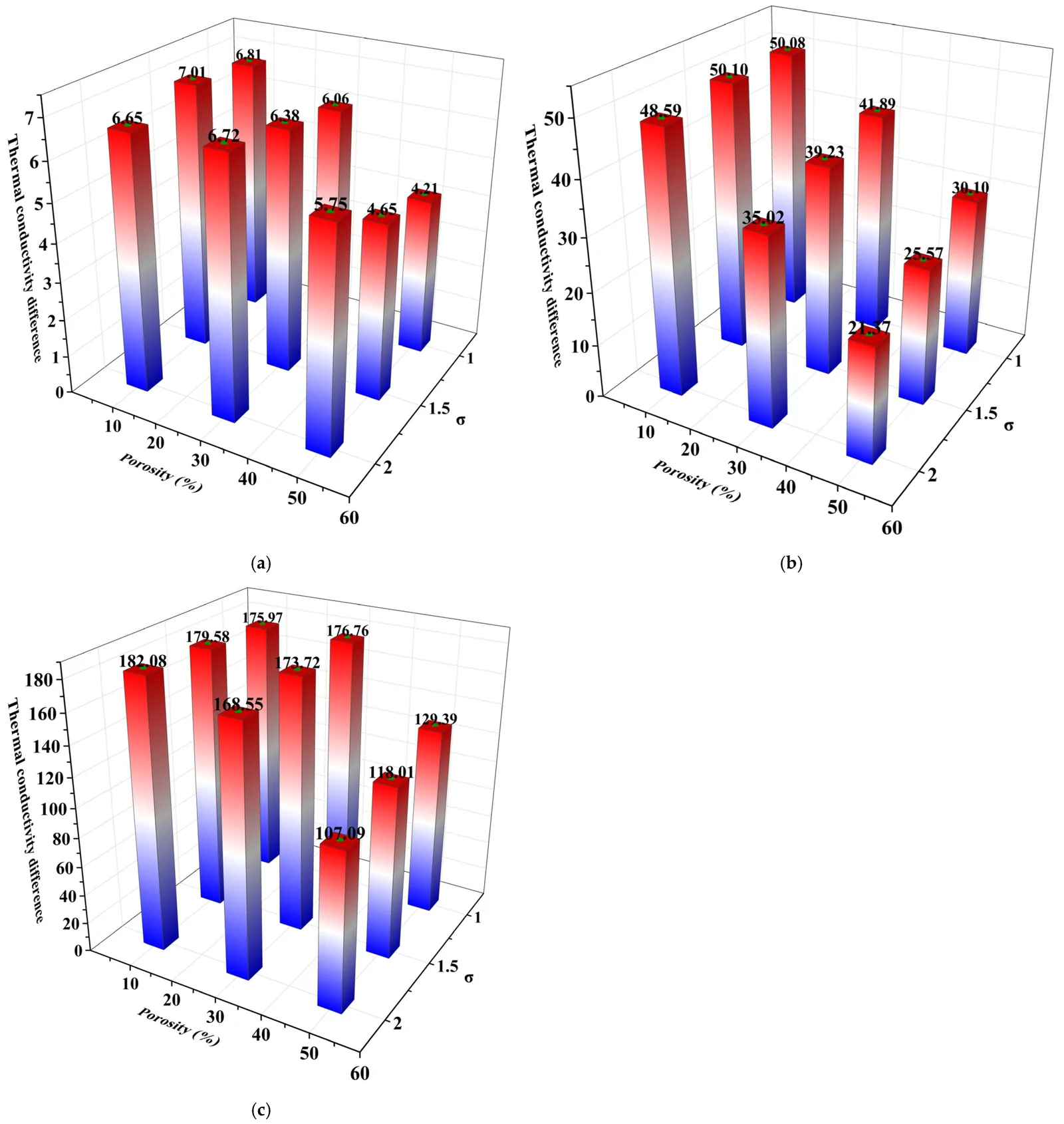
Bar charts of Δk value variation: (**a**) Diamond size 3, (**b**) Diamond size 5, (**c**) Diamond size 7.

However, from the combinations shown in the figures, it is evident that the magnitude of change in *k_eff_* is relatively small, making it difficult to discuss the coupled influence of diamond particle number and porosity. Therefore, Δ*_k_* is defined as the difference in *k_eff_* when the number of diamond particles increases from 27 to 729 for each combination, allowing for analysis of more combinations. Figure 6a–c present bar charts showing the variation of Δ*_k_* values for different combinations of σ and porosity, with diamond size fixed at 3, 5, and 7, respectively. It can be observed from the figures that Δ*_k_* increases significantly with increasing porosity.

This phenomenon arises because, in low-porosity systems, the silver matrix already forms a relatively continuous high-thermal-conductivity network. Introducing diamond reinforcement under these conditions can further enhance thermal conductivity, but the increase is relatively limited, as heat transport is already predominantly governed by the silver network. As porosity increases to 30% or even 50%, the continuity of the silver network is severely disrupted. In this scenario, increasing the number of high thermal conductivity diamond particles effectively constructs alternative thermal conduction pathways bridging the pores, producing an enhancement effect and significantly improving the efficiency of thermal conductivity increase.

**Figure 7:**
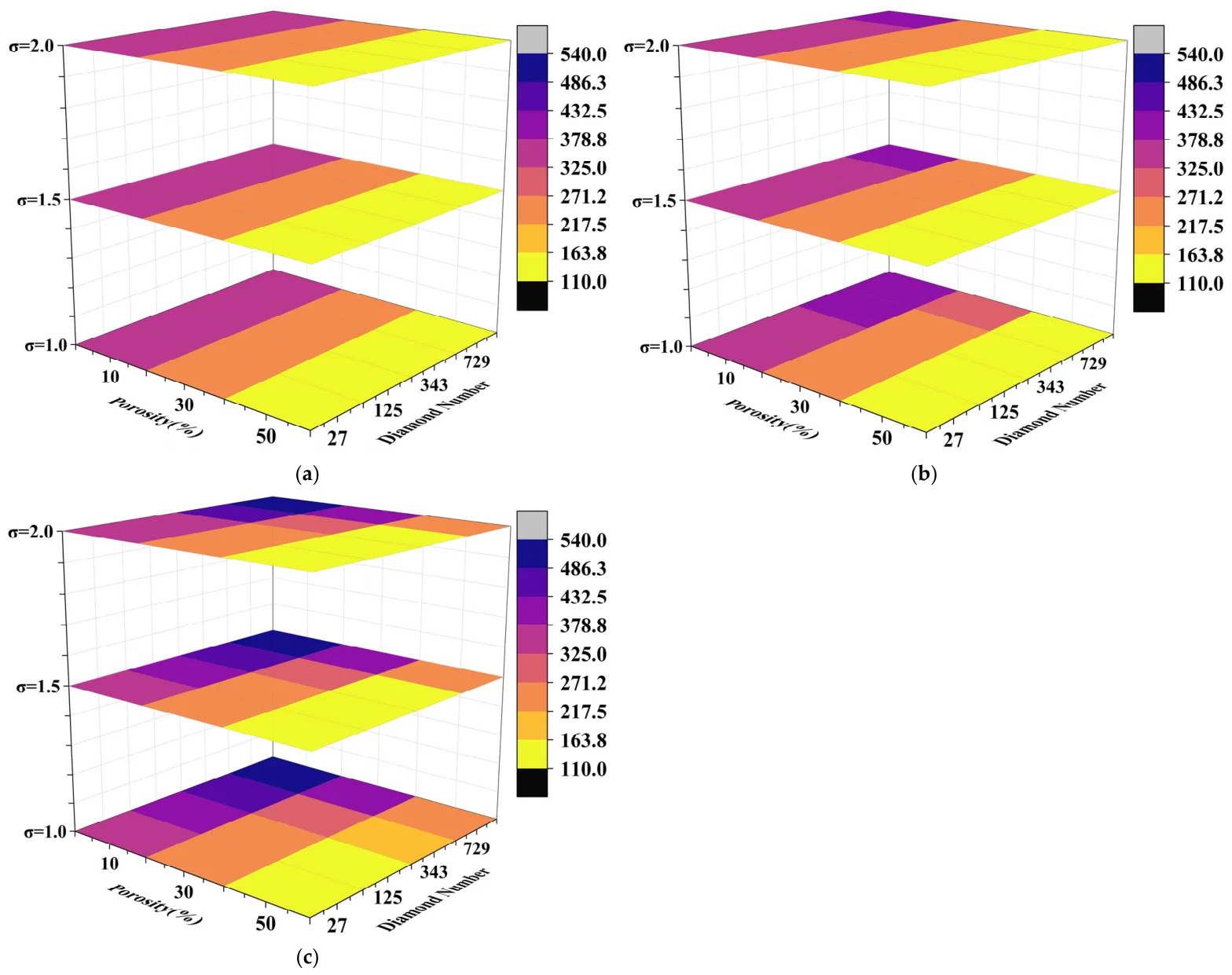
Heatmaps of the combined influence of porosity and diamond particle number on *k_eff_*: (**a**) Diamond size 3, (**b**) Diamond size 5, (**c**) Diamond size 7.

Figure 7a–c illustrate the combined influence of porosity and diamond particle number on *k_eff_*, with diamond size fixed from 3 to 7. It can be seen that *k_eff_* is higher when the combination of porosity and diamond particle number approaches low porosity and high diamond particle count. Although increasing the number of diamond particles can accelerate the efficiency of *k_eff_* improvement even at higher porosity levels, integrating the observations from Figure 5, Figure 6 and Figure 7 reveals that the enhancement effect of diamond particle number is constrained by porosity. At low porosity (10%), increasing the number of diamond particles has a more pronounced effect on improving *k_eff_*; whereas at high porosity (50%), even with an increased number of diamond particles, *k_eff_* remains limited by the blocking effect of pores.

### 4.3. Influence of Phase Morphology on Effective Thermal Conductivity

**Figure 8:**
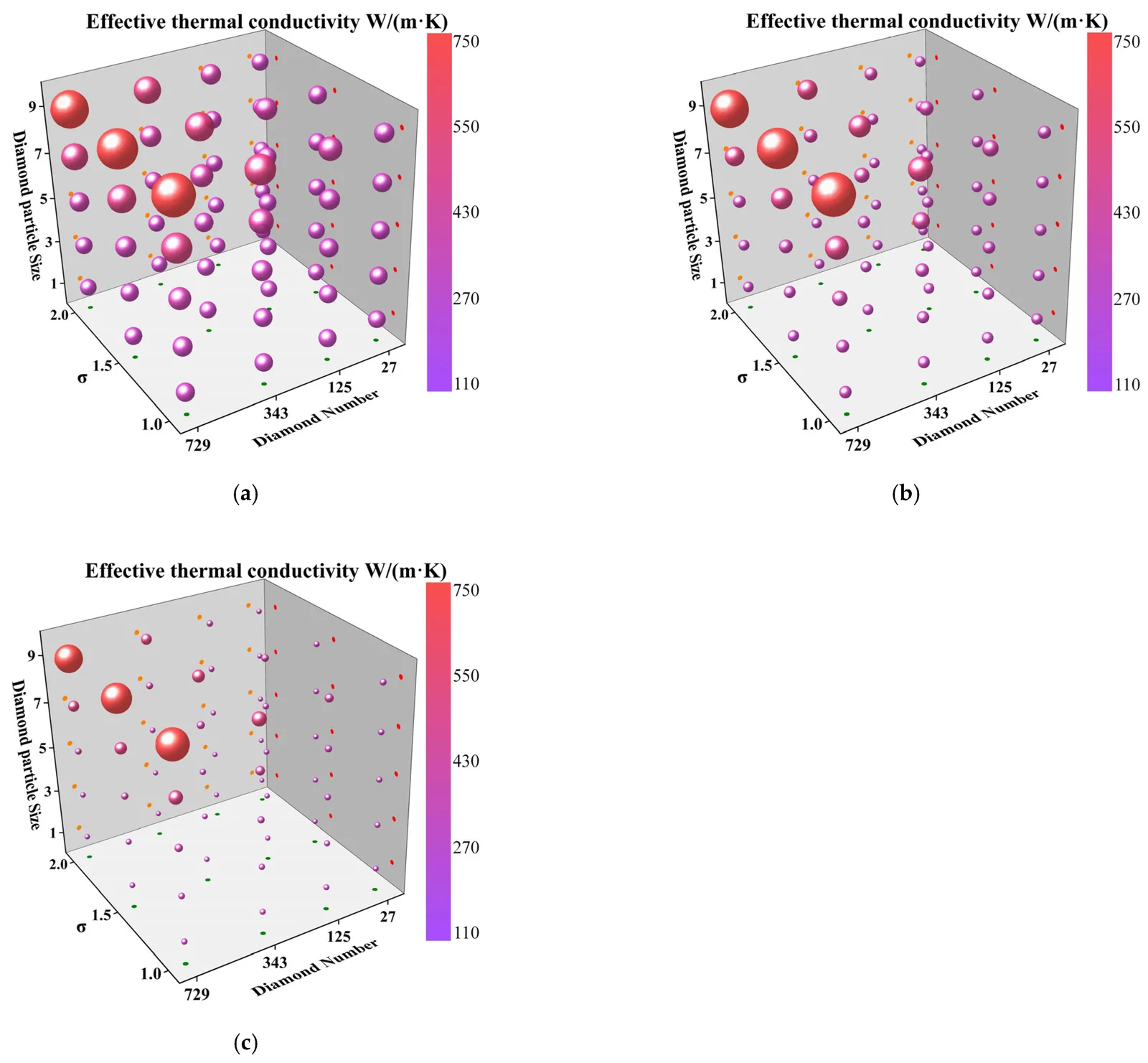
*k_eff_* values for different σ, diamond particle size, and diamond particle number parameter combinations under fixed porosity: (**a**) Porosity 10%, (**b**) Porosity 30%, (**c**) Porosity 50%.

It can also be observed from Figure 6 that the Δ*_k_* value increases significantly with increasing diamond particle size. To further analyze this phenomenon, the variation of *k_eff_* for different combinations of diamond particle size and diamond particle number is discussed while keeping the total diamond volume fraction approximately constant. Figure 8a–c summarize the *k_eff_* results for the 180 parametric RVEs under porosity conditions ranging from 10% to 50%. In the figures, larger sphere volume and colors closer to red indicate higher *k_eff_*. It can be seen that as diamond particle size increases, *k_eff_* exhibits a significant upward trend across all combinations, consistent with the conclusion drawn from Figure 6.

**Figure 9:**
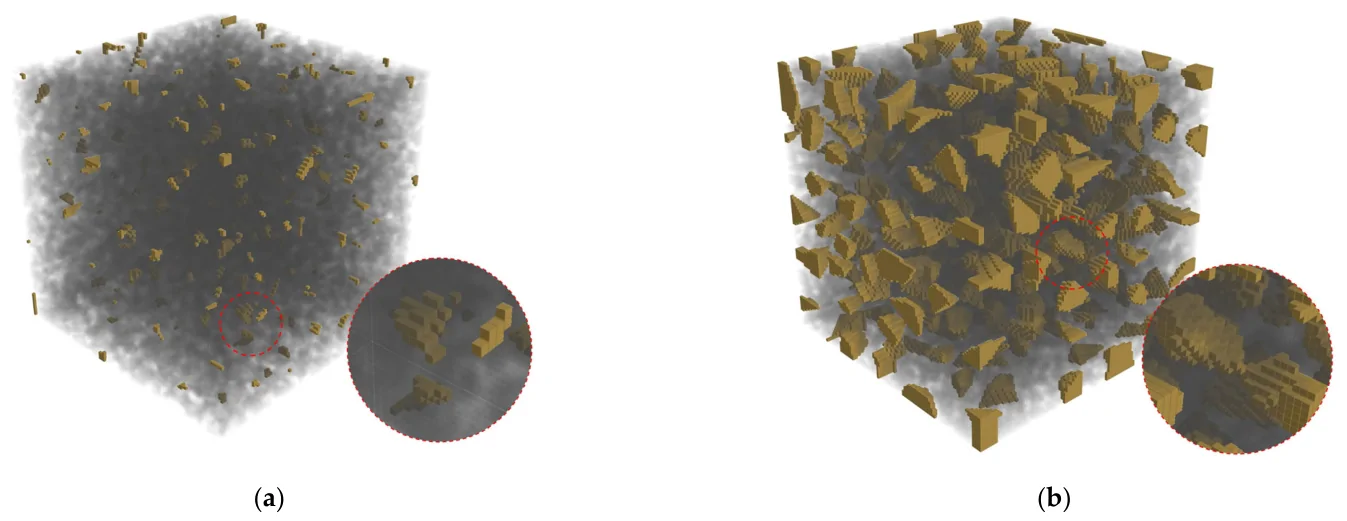
Details of RVE diamond particles: (**a**) Diamond size 3, (**b**) Diamond size 7.

The reason for this phenomenon is that larger particles more readily increase the probability of forming continuous high thermal conductivity pathways within the RVE. Furthermore, as illustrated in Figure 9, with increasing particle size, the probability of forming continuous or near-continuous heat transfer channels between high thermal conductivity particles increases. This reduces the distance heat flow must detour through low thermal conductivity phases (silver matrix and pores), thereby enhancing the equivalent heat transfer capability.

**Figure 10:**
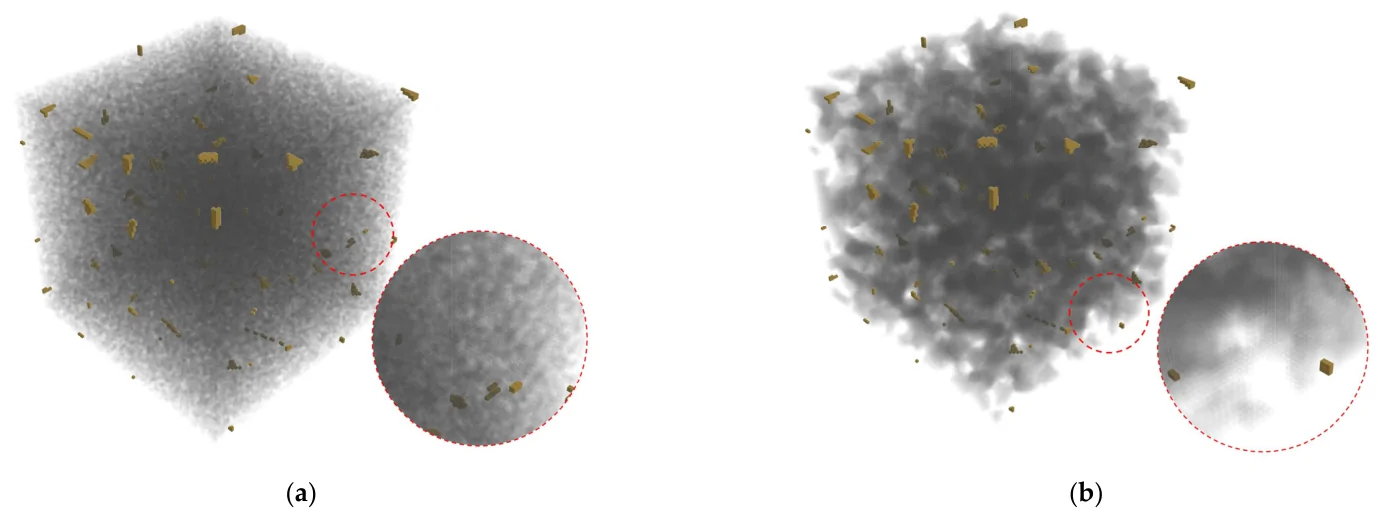
Details of RVE pore morphology: (**a**) σ = 0.5, (**b**) σ = 2.5.

Correspondingly, an increase in the pore spatial correlation length σ, which produces more spatially correlated and connected pore regions, results in a decrease in *k_eff_*. Simulation results show that, with other conditions fixed, increasing σ from 1.0 to 2.0 generally leads to a decreasing trend in *k_eff_* across all combinations. This phenomenon occurs because σ essentially represents the spatial correlation length of pores. A smaller σ indicates finer, more dispersed pores, allowing heat flow to bypass local defects within the silver matrix. A larger σ indicates more connected pore regions and a stronger pore-clustering tendency. Multiple pores merge spatially, forming large-scale low thermal conductivity regions that force heat flow to undertake long detours, thereby reducing *k_eff_*. Figure 10 shows the pore morphology of RVEs generated with different σ values.

**Figure 11:**
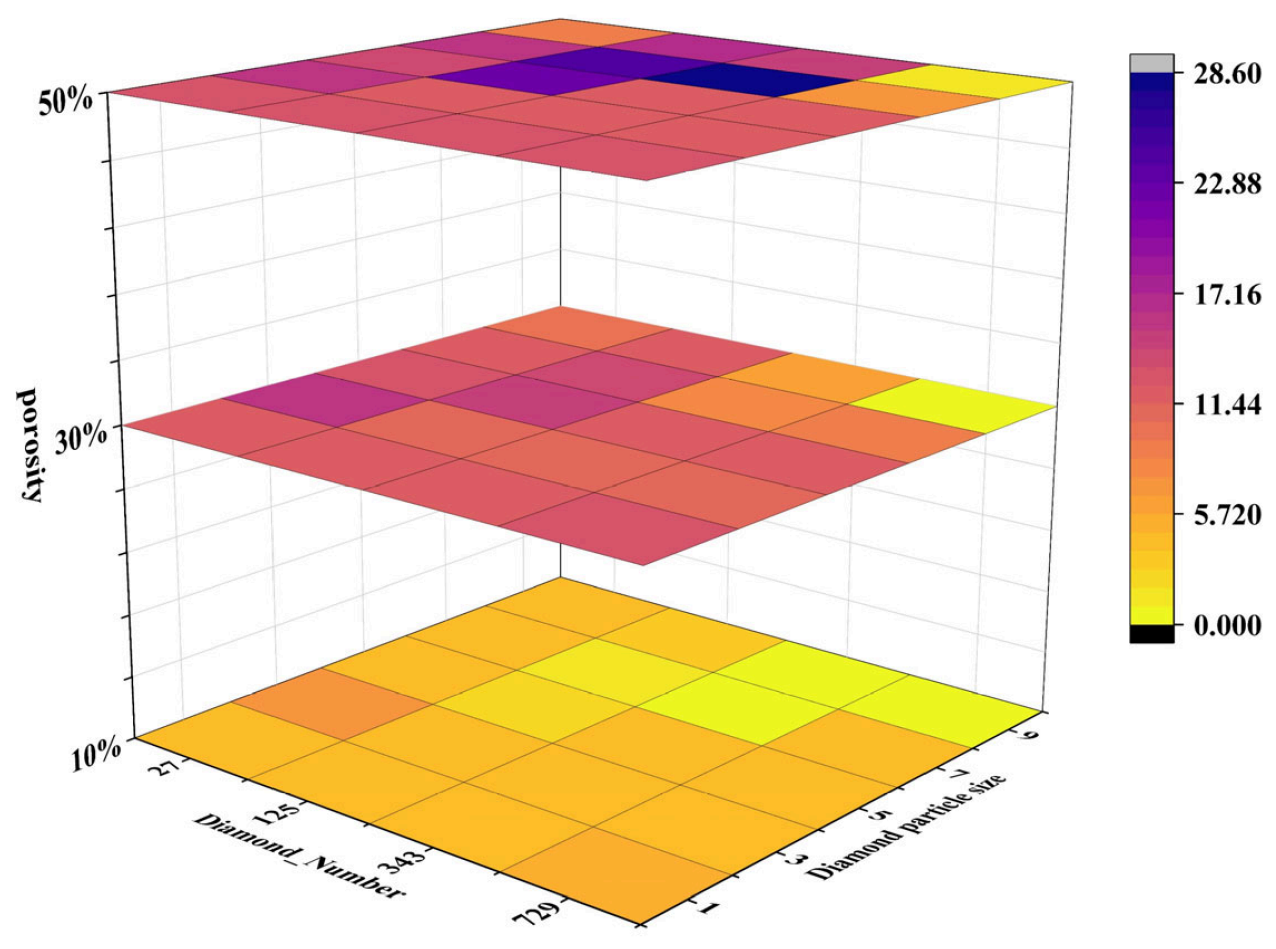
Heatmaps of $\Delta_{k}^{\prime }$ value variation.

Analysis of the data reveals that the magnitude of change in *k_eff_* is relatively small, making it difficult to discuss the coupled influence of σ with other parameters. Therefore, $\Delta_{k}^{\prime }$ is defined as the difference in *k_eff_* when σ is reduced from 2.0 to 1.0 for each combination, and all combinations are analyzed. Figure 11 presents heatmaps showing the variation of $\Delta_{k}^{\prime }$ values for different combinations of diamond particle number and diamond size under fixed porosity, with porosity ranging from 10% to 50%. It can be observed from the figures that the influence of σ variation on $\Delta_{k}^{\prime }$ is amplified as porosity increases.

The reason for this phenomenon is that under low-porosity conditions, the impact of pores on overall thermal conductivity is relatively weak. Even if changes in σ alter pore morphology, heat flow still primarily passes through the silver matrix, and the contribution of pore-morphology changes to enhancing thermal conductivity is small. As the volume fraction of pores increases, the probability of heat flow passing through pores increases, and the contribution of pore morphology to enhancing thermal conductivity gradually becomes more significant. However, compared to reducing σ, lowering porosity remains the primary factor in improving thermal conductivity.

**Figure 12:**
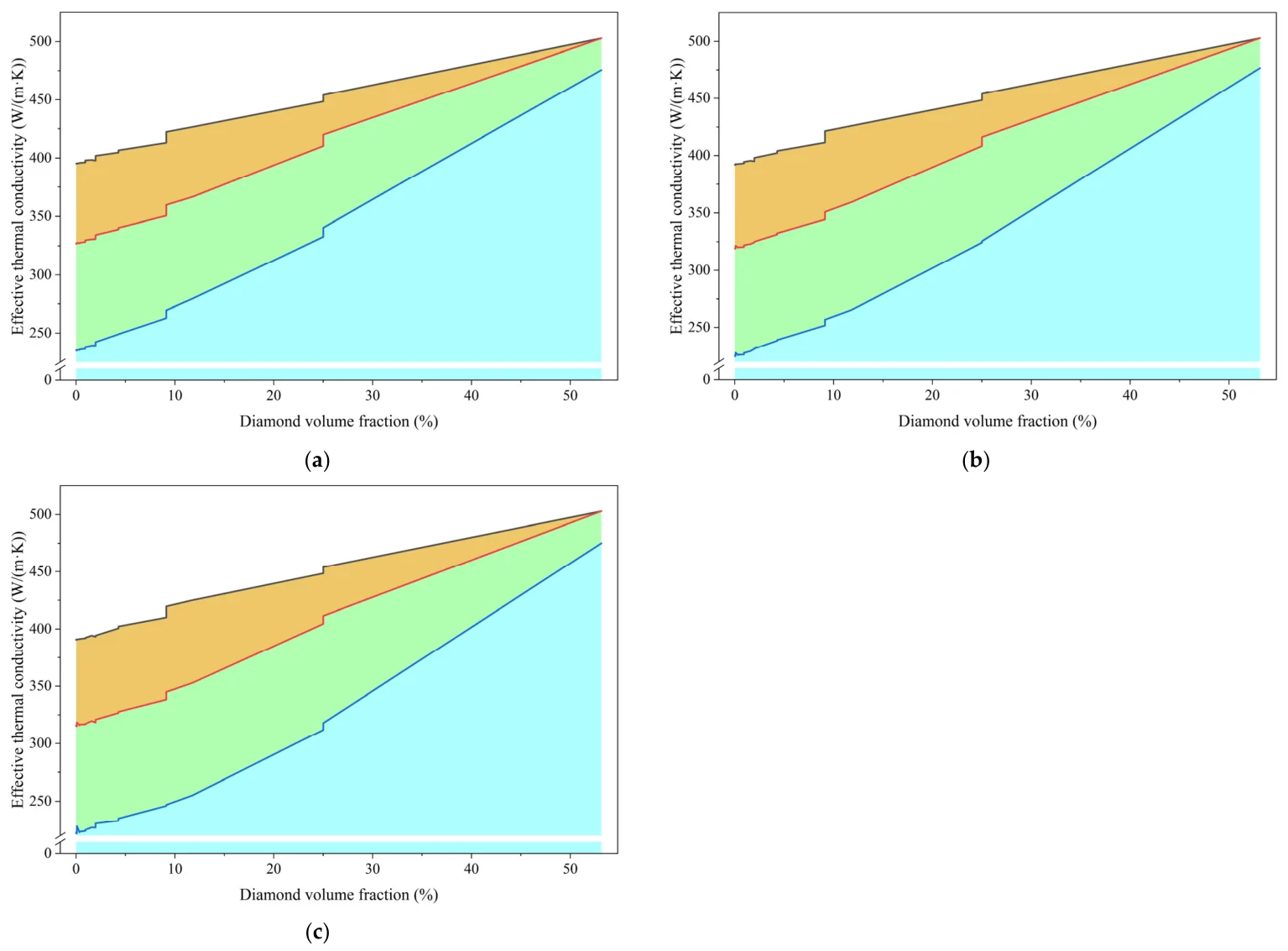
*k_eff_* variation with *ϕ_d_*: (**a**) σ = 1.0, (**b**) σ = 1.5, (**c**) σ = 2.0.

In engineering design and composite material characterization, the diamond reinforcement phase is typically represented by its volume fraction *ϕ_d_* as a key structural parameter. To ensure that the numerical results can be directly compared with engineering and experimental studies, this research employs Equation (14) to calculate *ϕ_d_*, where each given combination of diamond particle number and size parameters corresponds to a unique *ϕ_d_*. Figure 12a–c illustrate the trend of *k_eff_* as a function of *ϕ_d_* under different porosity levels, with σ ranging from 1.0 to 2.0, and the orange, green and blue areas in the figure represent porosity levels of 10 per cent, 20 per cent and 30 per cent respectively. As *ϕ_d_* increases, *k_eff_* across different porosity and σ combinations generally shows a clear upward trend, consistent with the conclusions previously drawn regarding particle number and particle size.

### 4.4. Sensitivity Analysis of Interface Defects

Considering that the bonding quality at the diamond/Ag interface can significantly affect the heat-transfer capability of the composite, an interface-defect descriptor was further introduced to supplement the analysis of interfacial imperfection effects. Since the present study has not established an experimentally calibrated model for the true interfacial thermal resistance, the geometric features of pores near the interface are not directly equated with the true interfacial thermal resistance. Instead, the global interfacial pore coverage ratio is adopted as a proxy to characterize the degree of interfacial imperfection. This parameter is defined by the following equation as the ratio of the number of interfacial facets where the diamond surface is directly adjacent to pores to the total number of interfacial facets on the diamond surface.

(31) $$\phi_{\mathrm{int},\mathrm{global}}=\frac{N_{d-p}}{N_{d-\mathrm{Ag}}+N_{d-p}}$$

where *N_d_*_−*p*_ denotes the total number of interfacial facets on the diamond surface that are directly adjacent to pores, and *N_d_*_−*Ag*_ denotes the total number of interfacial facets on the diamond surface that are directly adjacent to the silver phase. Correspondingly, the interfacial integrity coefficient can be defined as

*η*_int,global_ = 1 − *ϕ*_int,global_ (32)

A larger value indicates a higher degree of continuous contact at the diamond/Ag interface and, therefore, a more intact interface. A smaller value indicates a stronger pore-induced weakening effect near the interface and a more pronounced degree of interfacial imperfection.

**Figure 13:**
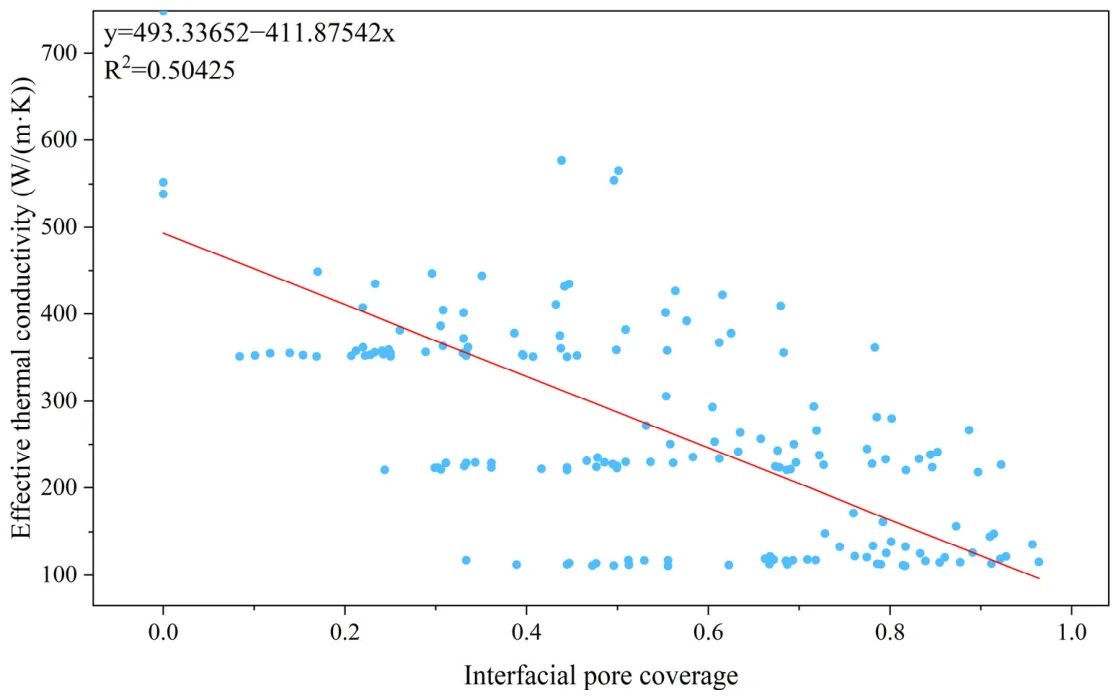
Sensitivity analysis of *k_eff_* to the global interfacial pore coverage ratio.

As shown in Figure 13, *ϕ*_int,global_ exhibits a clear negative correlation with *k_eff_*. As the global interfacial pore coverage increases, the effective thermal conductivity shows an overall decreasing trend, indicating that interfacial imperfection can significantly weaken the overall heat-transfer capability of the three-phase composite. Linear fitting further confirms a stable negative correlation between the two, with a Pearson correlation coefficient of −0.710 and a Spearman correlation coefficient of −0.613. These results indicate that, even without explicitly introducing interfacial thermal-resistance elements, the disruptive effect of pores near the interface on the continuity of heat-transfer pathways can still be effectively captured by the geometric proxy for interface defects.

Table 1 summarizes the statistics of interface defects grouped by the nominal porosity parameter. It can be seen that, as the nominal porosity parameter increases from 10% to 30% and 50%, the mean global interfacial pore coverage ratio increases from 0.282 to 0.542 and 0.715, respectively, while the mean effective thermal conductivity decreases from 408.333 to 281.285 and 156.526, respectively. These results indicate that an increase in pore content not only directly reduces the effective heat-conduction cross-sectional area of the continuous silver phase, but also further weakens interfacial heat transfer by increasing the degree of imperfection at the diamond/Ag interface.

From a physical point of view, when more pores surround the diamond surface, the effective contact area between the diamond and the silver phase is reduced, and heat transfer across the interface is weakened. As a result, the beneficial contribution of the highly conductive diamond particles to the overall heat-transfer network cannot be fully utilized. Therefore, within the parameter range considered in this study, interface defects can be regarded as an important supplementary factor affecting the effective thermal conductivity, and their effect is consistent in direction with the overall decrease in thermal conductivity caused by increasing porosity.

### 4.5. Quantitative Sensitivity Analysis

To avoid relying solely on images and trend plots for qualitative interpretation, a quantitative sensitivity analysis was further introduced in this study to formally rank the relative influences of different microstructural parameters on *k_eff_*. This analysis follows a data-driven “microstructural parameter–macroscopic response” mapping framework [28], in which the dominant factors under multi-parameter interactions are quantitatively identified through statistical learning and variance decomposition. In this work, the 180 RVE-FEM results were treated as a microstructure–macroscopic thermal-response database, on the basis of which a formal statistical ranking and data-driven cross-validation were performed.

In the present study, the nominal input parameters include the nominal porosity parameter *p*, the Gaussian filter width, the number of diamond particles DiaNum, and the particle size Grain size, while the macroscopic output variable is the effective thermal conductivity *k_eff_*. To compare the relative contributions of these input parameters to *k_eff_* from a statistical perspective, a Type-II analysis of variance (ANOVA) was first conducted on the nominal input parameters, and the effect size *η*^2^ was adopted as the sensitivity metric, defined as follows:

(33) $$\eta_{i}^{2}=\frac{SS_{i}}{SS_{\mathrm{total}}}$$

where *SS_i_* is the sum of squares associated with the *i*-th input parameter, and *SS*_total_ is the total sum of squares. This metric reflects the proportion of the total variance in the output variable explained by each parameter. A larger $\eta_{i}^{2}$ indicates a more significant influence of that parameter on *k_eff_*.

The Type-II ANOVA results show that the main-effect *η*^2^ values of the four nominal input parameters are 0.480 for *p*, 0.196 for Grain size, 0.116 for DiaNum, and only 0.001 for width. As shown in Table 2, within the data range considered in this study, the pore-related parameter exhibits the highest explanatory power for the effective thermal conductivity and is therefore the dominant factor; particle size and particle number show secondary but still significant effects; the contribution of the Gaussian filter width is the weakest. These results statistically support the trend-based conclusion presented earlier, namely that pore-related factors play the primary role among the parameters affecting *k_eff_*.

Furthermore, considering that the nominal input parameters do not correspond exactly one-to-one to the actually generated microstructures, a second-level analysis was performed using the measured structural descriptors obtained after generation. These descriptors include the actual pore volume fraction *f_p_*, the actual diamond volume fraction *f_d_*, the interface-defect descriptor *ϕ*_int_, and the Gaussian filter width (width). On this basis, a standardized linear regression model was constructed as follows:

*z*(*k*_eff_) = *β*_1_*z*(*f_p_*) + *β*_2_*z*(*f_d_*) + *β*_3_*z*(*ϕ*_int_) + *β*_4_*z*(width) + *ε* (34)

where *z*(⋅) denotes the standardized variable, *β_i_* denotes the standardized regression coefficient, and *ε* denotes the residual term. Since all input variables were standardized, the magnitude of |*β_i_*| can be used directly to compare the relative influences of different structural descriptors on *k_eff_*.

The regression results show that the standardized regression coefficient is *β* = −0.565 for the actual pore volume fraction, *β* = 0.442 for the actual diamond volume fraction, *β* = −0.118 for *ϕ*_int_, and only *β* = −0.008 for width, with a coefficient of determination of *R*^2^ = 0.998. These results indicate that, after controlling for the actual phase volume fractions, interface defects still exhibit an identifiable negative contribution, but their effect is weaker than that of the pore volume fraction and the diamond volume fraction. In other words, the actual pore volume fraction remains the primary structural factor governing the variation of *k_eff_*, while the diamond volume fraction provides a significant positive compensating effect. By contrast, interface defects mainly act as a secondary weakening factor, and the independent contribution of the Gaussian filter width itself is the weakest. These findings further show that the earlier conclusion that porosity dominates the variation of *k_eff_* is not only consistent in terms of trend, but is also repeatedly supported by formal statistical decomposition and data-driven cross-validation.

### 4.6. Robustness Analysis

Given that the parametric study in this work is carried out using a standard RVE size of 100 × 100 × 100 voxels, it is necessary to verify the representativity and statistical stability of this size in predicting the effective thermal conductivity. To this end, supplementary analyses were performed while keeping the original microstructure-generation logic, material parameters, and boundary conditions unchanged. Specifically, three representative porosity levels were selected, namely *p* = 10%, 30%, and 50%, while the Gaussian filter width, the number of diamond particles, and the particle size were fixed at width = 1.5, DiaNum = 125, and Grainsize = 5, respectively. On this basis, three RVE sizes, namely 80 × 80 × 80, 100 × 100 × 100, and 120 × 120 × 120, were constructed. For each size, five independent realizations were generated using different random seeds, in order to evaluate the effects of RVE size and realization-to-realization fluctuation on the effective thermal conductivity *k_eff_*.

**Figure 14:**
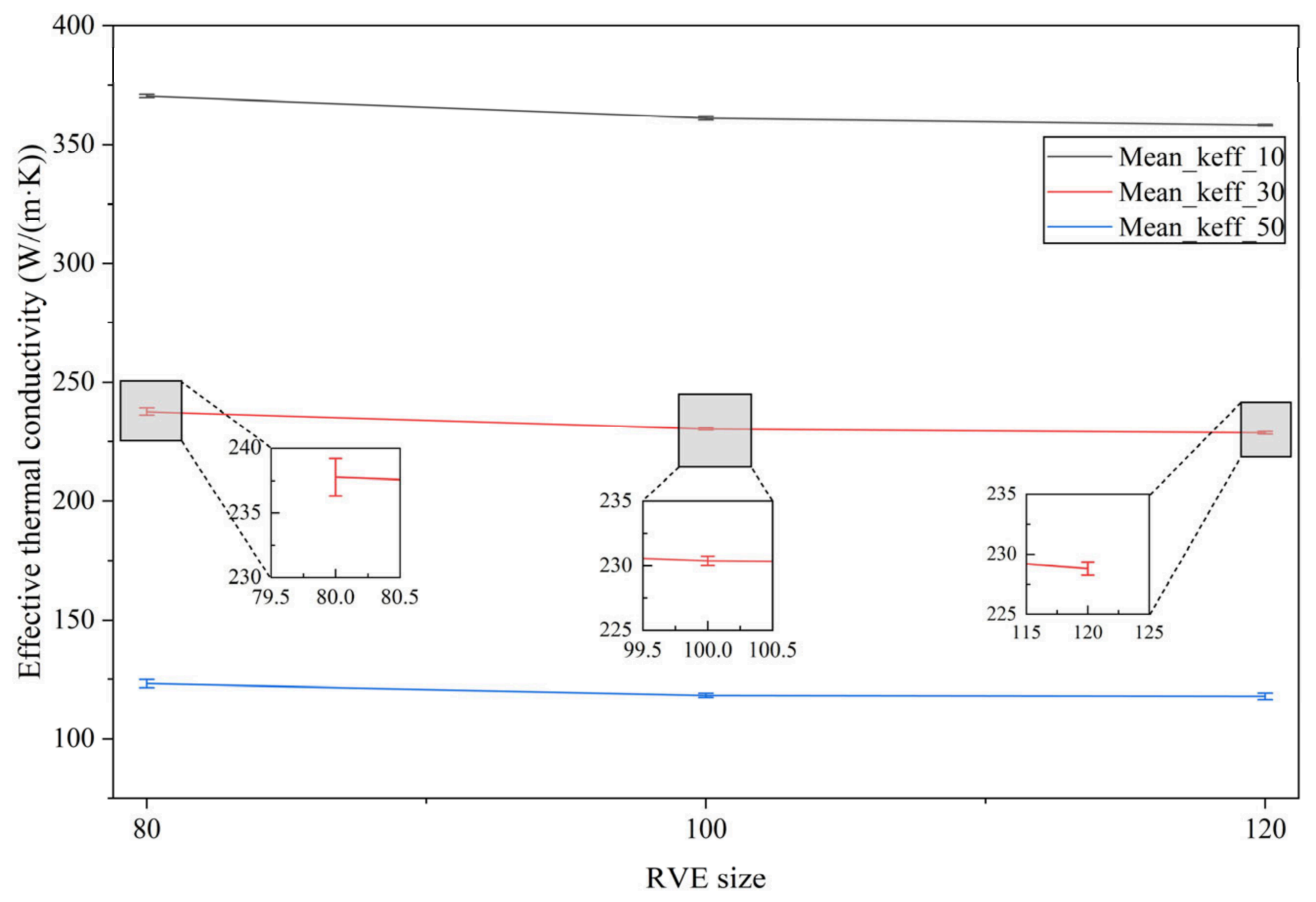
Mean effective thermal conductivity and standard deviation for representative cases at different RVE sizes. The three curves correspond to the representative low-, medium-, and high-porosity cases, respectively. The error bars denote the standard deviations of different random realizations under the same parameter combination.

To compare the statistical stability of the results at different sizes, the mean value, standard deviation, and coefficient of variation were calculated from the multiple realizations for each case at the same size. In addition, the results for 120 × 120 × 120 were taken as the reference, and the relative deviations of the smaller RVEs from this reference were evaluated. Figure 14 presents the variation of the mean *k_eff_* with RVE size for the three representative cases, where the error bars denote the standard deviations among different realizations. As shown in Figure 14, when the RVE size increases from 80 × 80 × 80 to 100 × 100 × 100, the mean *k_eff_* of all three cases becomes noticeably more stable; when the size is further increased to 120 × 120 × 120, the change in the mean value becomes much smaller, indicating that the size effect has been substantially reduced. Specifically, for the *p* = 10% case, the mean *k_eff_* values are 370.398, 361.081, and 358.080 W·m^−1^·K^−1^, respectively; for the *p* = 30% case, the corresponding values are 237.763, 230.386, and 228.785 W·m^−1^·K^−1^; and for the *p* = 50% case, they are 123.248, 118.334, and 117.937 W·m^−1^·K^−1^, respectively. These results show that the 80 × 80 × 80 RVE still exhibits a noticeable deviation from the larger-size reference solution, whereas the 100 × 100 × 100 RVE is already able to approximate the 120 × 120 × 120 reference results well.

Table 3 further summarizes the mean *k_eff_*, standard deviation, coefficient of variation, and the deviation relative to the 120 × 120 × 120 reference value for each representative case at different RVE sizes. As shown in Table 3, when the RVE size increases from 80 × 80 × 80 to 100 × 100 × 100, the relative deviations of the three cases decrease significantly from 3.440 to 4.504% to 0.337–0.838%. In particular, under the 100 × 100 × 100 condition, the deviations of the *p* = 10%, 30%, and 50% cases from the 120 × 120 × 120 reference values are only 0.838%, 0.700%, and 0.337%, respectively. Meanwhile, the corresponding coefficients of variation at 100 × 100 × 100 are 0.211%, 0.162%, and 0.767%, respectively, indicating that scatter among different random realizations remains at a low level.

In addition to the size effect, consistency across realizations is also an important criterion for evaluating model robustness. For the same set of nominal input parameters, the RVEs generated with different random seeds exhibit certain random differences in local particle positions and pore morphologies, but the corresponding effective thermal conductivity results remain overall concentrated and do not show systematic deviation. This indicates that, although the random microstructure-generation method adopted in this study is statistically stochastic, it still exhibits good stability and repeatability at the level of macroscopic thermal-conductivity response. In particular, under the 100 × 100 × 100 condition, the results obtained from different realizations for each case show good clustering, indicating that this size is not only representative in terms of the mean value, but also robust in terms of realization-to-realization variability.

Based on the above analysis, it can be concluded that, for the steady-state heat-conduction problem considered in this study, an RVE size of 100 × 100 × 100 provides a reasonable balance between computational accuracy and computational cost. On the one hand, this size is already sufficient to average out fluctuations caused by local microstructural randomness so that the predicted effective thermal conductivity becomes stable. On the other hand, its computational scale remains efficient enough for the subsequent large-scale parametric study. Therefore, the use of 100 × 100 × 100 as the standard RVE size throughout this work is justified, and this choice does not affect the main conclusions presented in Section 4.2 and Section 4.3 regarding the influences of porosity, diamond particle number, particle size, and Gaussian filter width on the effective thermal conductivity.

## 5. Conclusions

This study constructed an efficient three-dimensional random microstructure generation algorithm to create silver (Ag)-diamond-pore three-phase composite material RVEs with controllable phase distributions, aimed at predicting the macroscopic *k_eff_* of diamond-reinforced sintered silver composite thermal interface materials. Four key microstructural parameters were systematically investigated by categorizing them into two groups: phase content and phase morphology, to study their impact on the prediction results. The achievements and conclusions drawn from this study are as follows:

- The algorithm combines modified Voronoi segmentation, Gaussian filtering, and percentile-based phase separation techniques, achieving precise control over the size of the diamond reinforcement phase, porosity, and spatial distribution characteristics. The generated RVEs are suitable for subsequent finite element analysis to predict the effective thermal properties of the composite material.
- Phase content exerts a decisive influence on the composite’s *k_eff_*. Porosity shows a significant negative correlation with *k_eff_*. Taking a representative example with a diamond particle count of 27, a 40 percentage point increase in porosity resulted in an approximately 40.43% decrease in *k_eff_*. The enhancement effect of increasing the number of diamond particles on thermal conductivity is constrained by porosity; under low porosity conditions, increasing the number of diamond particles is more effective for improving thermal conductivity.
- Under conditions where the total volume fraction is kept approximately constant, diamond particle size exhibits a significant influence on *k_eff_*. As particle size increases, *k_eff_* generally trends upward. With other parameters fixed, an increase in σ leads to an overall decrease in *k_eff_*, and the influence of σ is amplified with increasing porosity. However, compared to adjusting pore morphology, reducing porosity remains the dominant factor in enhancing *k_eff_*.

## 6. Future Outlook

Although this study achieved the aforementioned results, it is limited by model simplifications and the current research stage. Factors such as voids, defects, and interfacial thermal resistance at the diamond-silver interface were not considered. The research conclusions are primarily applicable to cases with good interfacial bonding where pores are mainly distributed within the silver matrix. In real materials, interface quality is often a critical factor determining the performance of nanocomposites. Introducing interfacial void and interfacial thermal resistance models is a direction for future work. Concurrently, utilizing the RVE generator developed in this study to conduct batch finite element simulations in Abaqus, extending beyond steady-state heat conduction to include thermo-mechanical coupling, thermal cycling, and contact mechanics simulations, will enable the generation of large-scale, multi-modal datasets encompassing geometry/morphology, physical parameters, and responses (such as *k_eff_*, maximum stress, etc.).

Subsequently, this data will be used to train and compare multiple machine learning models and develop reliable surrogate models. These surrogate models should be capable of replacing some expensive simulations and experiments within acceptable error tolerances, thereby enabling rapid materials design and optimization. These expanded efforts are expected to elevate the current methodology from mechanistic exploration to an engineering-usable predictive tool, while also providing quantitative guidance for subsequent experimental validation and process optimization.

## Figures and Tables

**Table 1:** Statistical results of the interface-defect descriptor and *k_eff_* under different porosity parameters.

Porosity/%	Sample Size	Average Global Pore Coverage	Standard Deviation	Mean *k_eff_*
10	60	0.282	0.183	408.333
30	60	0.542	0.205	281.285
50	60	0.715	0.160	156.526

**Table 2:** Quantitative sensitivity statistics of microstructural parameters on the effective thermal conductivity.

Parameter	Sample Size	Average Global Pore Coverage
*p*	0.480	1
Grain size	0.196	2
DiaNum	0.116	3
width	0.001	4

**Table 3:** Statistical results of *k_eff_* at different RVE sizes. The table lists the mean effective thermal conductivity, standard deviation, coefficient of variation, and the deviation relative to the largest-size reference value for the three representative cases at different RVE sizes.

RVE Size	Porosity/%	Mean *k_eff_*	Std *k_eff_*	CV/%	Relative Deviation vs. 120^3^/%
80^3^	10	370.398	0.709	0.191	3.440
100^3^	10	361.081	0.763	0.211	0.838
120^3^	10	358.080	0.269	0.075	0.000
80^3^	30	237.763	1.441	0.606	3.924
100^3^	30	230.386	0.372	0.162	0.700
120^3^	30	228.785	0.564	0.246	0.000
80^3^	50	123.248	1.682	1.365	4.504
100^3^	50	118.334	0.908	0.767	0.337
120^3^	50	117.937	1.376	1.166	0.000

## Data Availability

The original contributions presented in this study are included in the article. Further inquiries can be directed to the corresponding authors.

## Abbreviations

The following abbreviations are used in this manuscript:

Ag	Silver
CTE	Coefficient of thermal expansion
CNF	Carbon nanofiber
DC3D8	Abaqus 8-node three-dimensional heat-transfer brick element
FEM	Finite element method
GaN	Gallium nitride
GRF	Gaussian random field
HEMTs	High-electron-mobility transistors
INP	Abaqus input file
*k_eff_*	Effective thermal conductivity
MOSFETs	Metal–oxide–semiconductor field-effect transistors
RVE	Representative volume element
TIM	Thermal interface material
vol%	Volume percent
